# The preferences of people with amyotrophic lateral sclerosis on riluzole treatment in Europe

**DOI:** 10.1038/s41598-023-49424-3

**Published:** 2023-12-15

**Authors:** Albert C. Ludolph, Harish Grandjean, Evy Reviers, Valentina De Micheli, Cosetta Bianchi, Leonardo Cardosi, Hermann Russ, Vincenzo Silani

**Affiliations:** 1 Department of Neurology, German Center for Neurodegenerative Diseases (DZNE), University of Ulm, Ulm, Germany; 2 Charles River Associates International, Zürich, Switzerland; 3 European Organization for Professionals and Patients with ALS (EUpALS), Leuven, Belgium; 4 Business Integration Partners S.p.A, Milan, Italy; 5 Zambon Biotech SPA, Bresso, Milan Italy; 6 Sirius Scientific Consulting AG, 8852 Altendorf, Switzerland; 7 Department of Neuroscience and Laboratory of Neuroscience, IRCCS Istituto Auxologico Italiano, Milan, Italy; 8 Department of Pathophysiology and Transplantation, Dino Ferrari Center, Università degli Studi di Milano, Milan, Italy

**Keywords:** Diseases, Health care, Medical research, Neurology

## Abstract

The Patient Preference Survey aims to understand unmet needs related to riluzole management in people with Amyotrophic Lateral Sclerosis (ALS) and to identify which characteristics of a new formulation could better match their preferences. The survey involved 117 people with ALS (PALS) treated with riluzole in four European countries. The dysphagic PALS were least satisfied with the riluzole tablet and oral suspension and with ease in self-administration; up to 46% of respondents postponed or missed the treatment due to swallowing difficulties and need of caregiver assistance. Overall, 51% of tablet and 50% of oral suspension users regularly crushed or manipulated riluzole, respectively; PALS who always manipulated riluzole showed low satisfaction with the formulation and considered the risk of choking and pneumonia the most worrisome event. The survey evaluated the driving factors in choosing/switching the therapy: 67% of PALS declared a low risk of choking. The research finally evaluated which attributes of a new formulation would be preferred: the most relevant were ease of use (4.3/5), convenient/portable packaging (4.0/5) and oral-dissolving properties without tongue motility (3.9/5). The Patient Preference Survey suggests that patients have several unmet needs and preferences that could be addressed by a different formulation, e.g. using oral film technologies.

## Introduction

Amyotrophic lateral sclerosis (ALS) is a rare and progressive, neurodegenerative disease. It is characterized by a loss of upper and/or lower motor neurons with heterogeneous features and more than 30 genes identified as causative (the pathology is quite homogeneous indeed being TDP-43 + in 98% of cases both familial or sporadic)^1–4^. In Europe, the annual incidence and prevalence range from 2.1 to 3.8 and 4.1 to 8.4, per 100,000 persons, respectively^5,6^. The mean age at onset of symptoms is 51 to 66 years^3–5^. The course of ALS ends with respiratory failure and death, with a median survival rate of 2–4 years after onset^4^.

After the first clinical symptoms (e.g. muscular weakness, twitches or cramps), degeneration of the thoracic and respiratory muscles' motor neurons leads to problems in daily activities. People with ALS (PALS) become increasingly dependent on caregiver support, including the administration of treatment^7–9^. This significantly affects their quality of life (QoL) and creates a substantial socioeconomic burden^10,11^.

A cure for ALS is not available yet, and riluzole is the only approved Disease Modifying Treatment in Europe^12–14^. To date, riluzole is available in two formulations: tablets (50 mg) and oral suspension (5 mg/mL in 300 mL bottles). An independent meta-analysis of observational studies showed that riluzole leads to a broad spectrum of outcomes; in certain survival studies, the median survival increased to up to 19 months compared to placebo^15^. Thanks to long-term evidence, it is recommended that riluzole be administered as early as possible after diagnosis^16^ and maintained long-term to slow the progression of the disease in PALS^15,17^.

Adherence to riluzole treatment, whether with a tablet or liquid formulation, seems to be directly related to the progression of the disease and the onset of dysphagia. Therefore, the need for a riluzole formulation that may allow for better continuity of administration and thereby the best possible therapeutic effect on ALS disease progression, is an unresolved issue^18^.

A common strategy is to crush and then mix tablets with food before switching to oral suspension^19^. However, riluzole is approved to be administered 1 h before or 2 h after meals, precisely to avoid reduced adsorption due to the food effect^20^. It is known that crushing tablets can lead to medication errors, incomplete dosing, and decreased drug performance. It can also cause aspiration pneumonia and silent aspiration^21^, especially with foods, like yogurt^20^.

The liquid formulation of riluzole, which can be swallowed or administered via percutaneous endoscopic gastrostomy tubes, aims to improve adherence to the treatment by ensuring sufficient bioavailability and effectiveness of the medication^21^. Nevertheless, oral suspension also presents several challenges: it has a local anesthetic effect and a metallic taste, which are rather unpleasant. Moreover, managing multiple-dose glass bottles is more cumbersome than handling tablets. Finally, there is a perception that oral liquids are intended ‘for children’ because of the consistency of light cream^21^.

Taking into account these challenges, many new formulations, such as oral thin film, have been introduced in the US and European markets for therapeutic benefits^22^. They offer several advantages that might be useful for riluzole treatment and solve some of the challenges mentioned above. We conducted “Patient Preference Survey” (PPS) to better understand the unmet needs of PALS, their experience with the different riluzole formulations, and their satisfaction with them during the course of the disease. Considering the experiences of PALS with the currently available oral formulations, our goal was to evaluate their preferences regarding the properties of a new oral film formulation.

## Results

### Patient characteristics and treatment history

The study involved a total number of 117 participants: 58 were PALS and 59 were caregivers who responded on behalf of PALS unable to speak or use a computer autonomously. Since the caregivers reported the PALS’ preferences hereafter we use the term PALS to indicate all the respondents. 32 participants originated from Germany, 32 from Italy 49 from Spain and only 4 from France (unsuccessful recruitment).

To evaluate the reliability of the survey, 8 participants (2 per country) were asked to complete an online survey and to take part in a telephone qualitative interview. The test confirmed that the survey questions were comprehensible and appropriate in length and number.

Hereafter, this paper reports the results of the online survey which was completed by 109 participants.

The age of the respondents ranged between 30 and 70 years, and the average time from symptom onset to diagnosis was 12.5 months, 62% had a spinal onset type ALS (n = 68). Overall, 70% of PALS (n = 76) suffered from dysphagia and had problems eating (ranging from less severe form like choking during eating to complete dependence on feeding tubes). The baseline characteristics of the participants, including ALS onset and stages of ALS, are summarized in Supplementary Table 1.

Regarding their treatment history, 99% of PALS (n = 108) responded that they were on ALS-specific medications. At the time of the interview, 67% (n = 73 of patients had been treated with riluzole tablets, 29% (n = 32) with riluzole oral suspension, and 5% (n = 5) with edaravone) (Supplementary Table 2).

### Mean Satisfaction with the current riluzole formulations

PALS were moderately satisfied with riluzole tablets (3.2/5, where 5 is the maximum score) and oral suspension (3.0/5) with no statistical difference between formulations (*p* = 0.385) (Table 1). Tablet users were less satisfied with the formulation itself (3.0/5) and with the ease of self-administration (3.1/5). Oral suspension users did not appreciate manageability (2.9/5), ease of self-administration (2.7/5) and, interestingly, the formulation itself (2.9/5) (Table 1).

**Table 1:** Summary of Descriptive Statistics for mean satisfaction with current formulation according to tablet/oral suspension users.

Variable	Tablet user (n = 72)	Oral suspension user (n = 32)	Dysphagic (n = 76)	Non dysphagic (n = 33)	All PALS (n = 109)
Satisfaction with the current formulation (Average)					
Mean ± SD	3.2 ± 1.2	3.0 ± 1.1	2.9 ± 1.2	3.7 ± 1.0	3.1 ± 1.2
*p*-Value †	0.3846		0.0013		
Satisfaction with the formulation					
Mean ± SD	3.0 ± 1.4	2.9 ± 1.4	2.8 ± 1.4	3.4 ± 1.3	3.0 ± 1.4
Satisfaction with handiness and easiness of preparing the treatment before administration					
Mean ± SD	3.2 ± 1.4	2.9 ± 1.2	2.9 ± 1.3	3.7 ± 1.3	3.1 ± 1.4
Satisfaction with easiness to self-administer					
Mean ± SD	3.1 ± 1.4	2.7 ± 1.3	2.6 ± 1.4	3.7 ± 1.3	2.9 ± 1.4
Satisfaction with package weight and size					
Mean ± SD	3.4 ± 1.4	3.6 ± 1.2	3.1 ± 1.4	4.0 ± 1.0	3.4 ± 1.3

Level of satisfaction with the current formulation according to all participants surveyed (N = 109). Scale from 1 (not satisfied at all, left side) to 5 (greatly satisfied, right side). Categorical variables are summarized as percentage and absolute frequency versus the n° of non-missing observations [% (n/N)]; continuous variables are summarized as Mean, SD, n° of non-missing observations. † Nonparametric Mann–Whitney U-test. PALS: people with ALS; SD: standard deviation.

Overall, the mean satisfaction was significantly lower for patients affected by dysphagia than for those not affected (2.9/5 vs. 3.7/5 respectively; *p* = 0.001) (Table 1).

### Manipulating riluzole tablets

**Figure 1:**
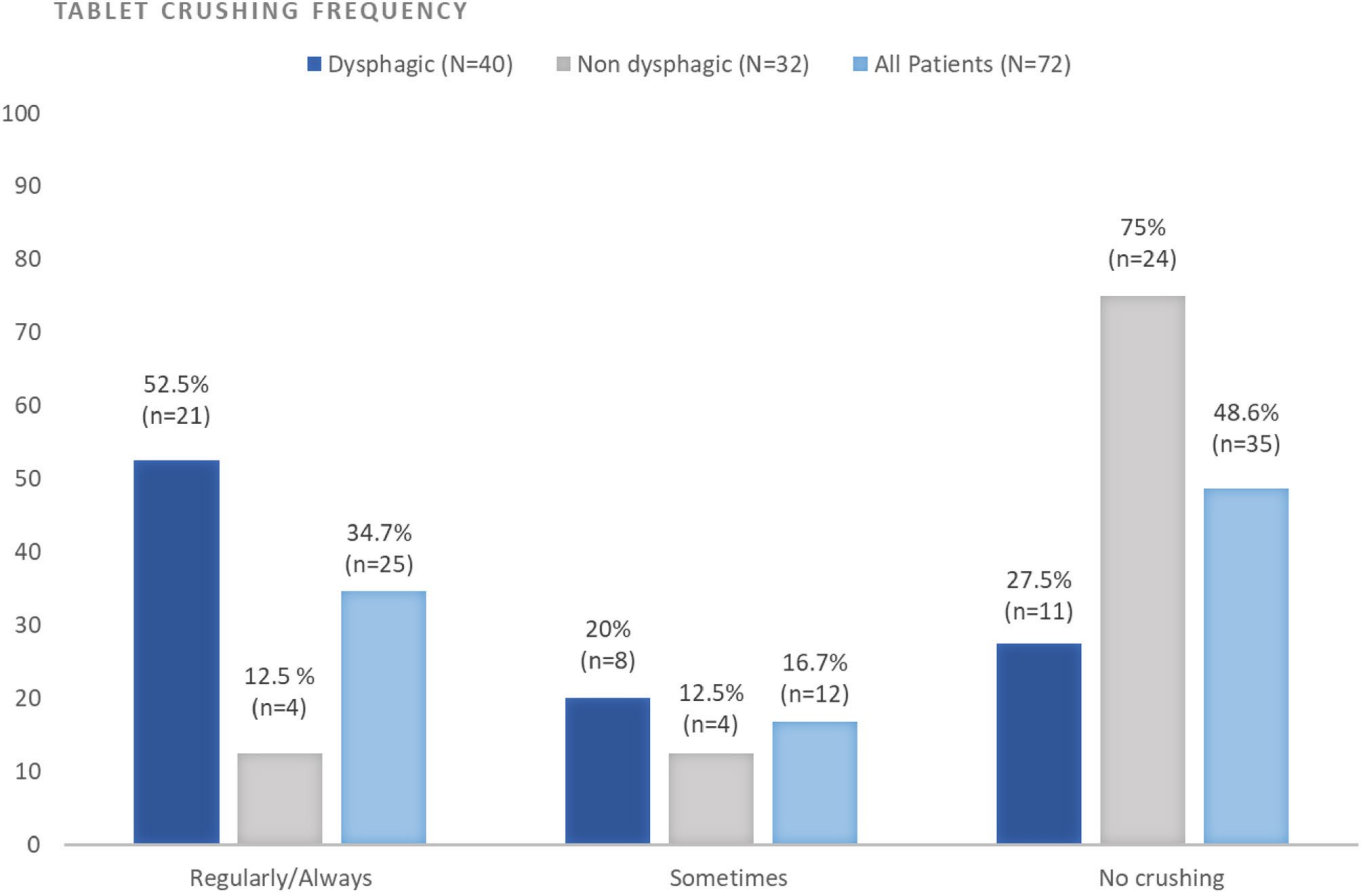
Frequency of manipulation of riluzole tablets by PALS. Current tablet manipulation. All percentages were calculated versus the answers per each group (dysphagic, non dysphagic, overall).

Due to difficulties in swallowing, 51% of tablet users (n/N = 37/72) declared that they sometimes or regularly crushed the tablets. Among dysphagic tablet users, up to 73% (n/N = 29/40) sometimes or regularly crushed the tablets compared to 25% (n/N = 8/32) in non dysphagic tablet users (*p* < 0.001) (Fig. 1). Notably, 19% of patients noticed some residue of crushed tablets that had not been ingested. Crushed tablets were mixed with food or liquid in 27% (n/N = 10/37) and 22% (n/N = 8/37), respectively. (Supplementary Fig. 1) These findings suggested that riluzole tablets manipulation is a widely used practice, regardless of the disease progression (i.e. the presence or absence of dysphagia). It should be noted that these practices may result in underdosing and could negatively impact patient satisfaction.

PALS who always crushed the tablets (35%, n/N = 25/72) showed a significantly lower mean satisfaction than those who never crushed the tablets (2.1/5 regularly/always crushing vs. 3.9/5 no crushing; *p* < 0.0001), while the mean satisfaction of PALS who sometimes crushed the tablets was 3.5/5 (*p* = 0.1363 vs no crushing) (Table 2).

**Table 2:** Level of satisfaction according to the frequency of alteration of formulation: tablet users.

Variable	Regularly/Always (n = 25)	Sometimes (n = 12)	No crushing (n = 35)	All patients (n = 72)
Satisfaction with the current formulation (Average)				
Mean ± SD	2.09 ± 1.04	3.5 ± 0.38	3.87 ± 1.02	3.19 ± 1.25
*p*† (regularly vs. no crushing)	< 0.0001			
*p*† (sometimes vs. no crushing)		0.1363		
Satisfaction with the formulation				
Mean ± SD	2.2 ± 1.22	3.58 ± 0.67	3.46 ± 1.36	3.04 ± 1.36
Satisfaction with handiness and easiness of preparing the treatment before administration				
Mean ± SD	2.04 ± 1.24	3.33 ± 0.89	3.97 ± 1.15	3.19 ± 1.43
Satisfaction with easiness to self-administer				
Mean ± SD	2 ± 1.12	3.25 ± 0.75	3.91 ± 1.25	3.14 ± 1.42
Satisfaction with package weight and size				
Mean ± SD	2.12 ± 1.27	3.83 ± 0.72	4.14 ± 1.03	3.39 ± 1.42

Categorical variables are summarized as percentage and absolute frequency versus the n° of non-missing observations [% (n/N)]; Continuous variables are summarized as Mean, SD, n° of non-missing observations. † Nonparametric Mann–Whitney U-test.

### Manipulating riluzole oral suspension

Among PALS treated with riluzole oral suspension, 50% (n/N = 16/32) declared that they sometimes or regularly manipulate the liquid formulation. In this subgroup, 69% of cases mixed the suspension with liquids or food (n/N = 11/16). Overall, the oral suspension was administered with a syringe (16%, n/N = 5/32), with a spoon (19%, n/N = 6/32) and through a feeding tube (22%, n/N = 7/32). It is noteworthy that in the qualitative interview, all the respondents stated that mixing with other liquids or food could facilitate administration due to the thick consistency of the riluzole oral suspension. Among oral suspension users (n = 32), PALS who always mixed the oral suspension (28%, n = 9) reported a mean satisfaction of 2.6/5 compared to 3.3/5 for PALS who never altered the oral suspension (*p* = 0.093). In particular, patients who regularly manipulate oral suspension showed low satisfaction with the formulation (2.0/5), with the easiness of self-administration, and with preparing the treatment before administration (2.4/5 and 2.8/5) as well as moderate satisfaction with package weight and size (3.3/5) (Table 3).

**Table 3:** Level of satisfaction according to the frequency of alteration of formulation: oral suspension users.

Variable	Regularly/always (n = 9)	Sometimes (n = 7)	No alteration (n = 14)	Other (n = 2)	All patients (n = 32)
Satisfaction with the current formulation (average)					
Mean ± SD	2.6 ± 1	3.1 ± 1.0	3.3 ± 1.1	2.5 ± 2.1	3.0 ± 1.1
*p*† (regularly vs. no alteration)	0.0934				
*p*† (sometimes vs. no alteration)		0.7354			
Satisfaction with the formulation					
Mean ± SD	2 ± 1	3 ± 1.3	3.4 ± 1.3	2.5 ± 2.1	2.9 ± 1.4
Satisfaction with handiness and easiness of preparing the treatment before administration					
Mean ± SD	2.8 ± 1.4	3 ± 1	3.1 ± 1.2	2.5 ± 2.1	2.9 ± 1.2
Satisfaction with easiness to self-administer					
Mean ± SD	2.4 ± 1.51	2.9 ± 1.21	2.7 ± 1.3	2.5 ± 2.1	2.7 ± 1.3
Satisfaction with package weight and size					
Mean ± SD	3.3 ± 1.1	3.6 ± 1.1	3.9 ± 1.1	2.5 ± 2.1	3.6 ± 1.2

Categorical variables are summarized as percentage and absolute frequency versus the n° of non-missing observations [% (n/N)]; Continuous variables are summarized as Mean, SD, n° of non-missing observations. † Nonparametric Mann–Whitney U-test.

### Postponing or missing a treatment

Postponing or missing the treatment is a possible consequence of low satisfaction. PPS asked all PALS if these events occurred and the rationale behind their conduct. In the overall population, 46% of the respondents (n/N = 50/109) either postponed or missed a daily treatment (n = 24 postponed and missed, n = 22 postponed, n = 4 missed) because of swallowing difficulties or a lack of independence in self-administration.

### Switching, interrupting or choosing ALS-specific treatments

13% of the respondents who were on ALS-specific medication either switched or interrupted their treatment (n/N = 14/108); among these, 8 PALS reported the reason for switching: 50% (n/N = 4/8) reported difficulties in swallowing the tablets, and 63% (n = 5/8) mentioned a variety of other reasons unrelated to the formulation (i.e. side effects, disease progression). (Supplementary Table 2) In the overall population, the main criteria for a possible future treatment switch were the concern of choking and, to the same extent, a doctor’s recommendation or ease of ingesting. (Supplementary Fig. 2).

Consistently, the most important criterion for choosing a new treatment was a low risk of choking (67% of patients n/N = 73/109). (Supplementary Fig. 3).

### Driving factors for choosing a different formulation

Considering the difficulties expressed by PALS taking riluzole tablets or the oral suspension formulation and the many strategies used to overcome them, PPS evaluated how the inherent characteristics of a new oral film formulation could improve satisfaction and adherence and decrease the overall burden on PALS. The most appreciated characteristics (mean scores) were ease of use and convenient packaging (4.3 and 4.0/5, respectively). The PALS also gave high scores for better self-administration (3.9/5), good oral-dissolving properties without tongue motility and no need for water or salivary stimulation (3.9/5 and 3.8/5 respectively). The survey results revealed mean scores above 3.0/5 also for lower risk of underdosing and lower risk of microbial contamination (3.8/5 and 3.7/5 respectively), followed by the reduction of metallic taste (3.7/5). Table 4 shows the results for tablets and oral suspension users, with no numerically relevant differences between the subgroups.

**Table 4:** Desired characteristics of a riluzole formulation expressed by all participants surveyed and analyzed overall and per formulation used.

Variable	Tablet user (n = 72)	Oral suspension user (n = 32)	Non dysphagic (n = 33)	Dysphagic (n = 76)	All patients (n = 109)
Driving factors for the choice new hypothetical product (average)					
Mean ± SD	3,8 ± 1.0)	3,9 ± 0,9	3,7 ± 0,9 )	4.0 ± 1.0 )	3,9 ± 1.0
Compellingness: It can dissolve in the mouth without the need to engage the tongue					
Mean ± SD	3,9 ± 1,4	4,00 ± 1,50	3,8 ± 1,4	4,00 ± 1,4	3,9 ± 1,4
Compellingness: No need for water and no salivary stimulant					
Mean ± SD	3,7 ± 1,5	4.0 ± 1,4	3,5 ± 1,5	4.0 ± 1,5	3,8 ± 1,5
Compellingness: Reduced risk of contamination* compared with available treatments					
Mean ± SD	3,6 ± 1,4	3,7 ± 1,1	3,3 ± 1,3	3,8 ± 1,3	3,7 ± 1,3
Compellingness: Reduced risk of underdosing** compared with available treatments					
Mean ± SD	3,7 ± 1,4	4,00 ± 1, 2	3,4 ± 1,2	4.0 ± 1,3	3,8 ± 1,3
Compellingness: Reduced metallic taste compared with available treatments					
Mean ± SD	3,6 ± 1,4	4,00 ± 1,2	3,3 ± 1,5	3,9 ± 1,2	3,7 ± 1,3
Compellingness: Potential self-administration and independence					
Mean ± SD	3,9 ± 1,4	3, 9 ± 1,50	4,0 ± 1,24	3,9 ± 1,5	3,9 ± 1,4
Compellingness: Intuitive and easy use without the need for extensive instruction					
Mean ± SD	4,3 ± 1.0	4,1 ± 1,4	4,4 ± 0,90	4,2 ± 1,2	4,3 ± 1,10
Compellingness: Convenient and portable packaging					
Mean ± SD	4,00 ± 1,2	3,8 ± 1,1	3,8 ± 1,4	4,0 ± 1,1	4.0 ± 1,2

Characteristics were based on typical oral film properties. Scale from 1 (not at all appealing) to 5 (very appealing). Categorical variables are summarized as percentage and absolute frequency versus the n° of non-missing observations [% (n/N)]. Continuous variables are summarized as Mean, SD, n° of non-missing observations.

These findings suggested that the manageability of the treatment, the ease of self-administration and the oral-dissolving properties could be important driving factors when choosing a treatment, influencing adherence. The average level of compellingness was numerically similar between dysphagic and non-dysphagic PALS or according to the frequency of formulation manipulation (Supplementary Table 3). Taken together, these results indicated that the features of a new hypothetical product (including reducing underdosing or contamination) may be appreciated during all phases of ALS.

## Discussion

PPS collected experiences and opinions from a representative group of PALS from four European countries. Although France’s contribution was smaller than that of the other countries due to recruitment issues, all the respondents were included in the analysis due to the descriptive nature of the study. The study involved PALS registered in patient associations dedicated to ALS as well as in ALS support groups and represents a heterogeneous setting in terms of disease duration. Patient characteristics are in line with previous epidemiological data^19,21^.

PPS showed that PALS have relevant unmet needs that cannot be fully satisfied with the riluzole tablet and oral suspension formulations currently available. The analysis of average satisfaction with the current formulations (both tablet and oral suspension) revealed PALS are moderately satisfied; among the respondents, dysphagic patients reported the lowest mean satisfaction score, indicating that the onset of dysphagia significantly and negatively influences acceptance of current riluzole formulations. This study also showed that, due to swallowing difficulties or lack of independence, more than 46% of PALS postpone or miss a treatment; therefore, dysphagia onset may also have a significant impact on treatment adherence.

PPS showed how difficulties with swallowing may lead to the manipulation of both tablets and oral suspension. In fact, PALS systematically altered the formulation of riluzole, crushing the tablets or mixing the oral suspension with other liquids. 51% of the tablet users crushed tablets to facilitate swallowing. Notably, 19% of them observed residual fragments from the crushed tablet that were not ingested thereby suggesting a potential risk of underdosing when PALS manipulated the tablets. Although oral suspension is formulated to improve adherence in case of severe dysphagia, 50% of oral suspension users reported that they still manipulated their medication. The viscous liquid and its large volume were considered to be moderately bothersome. This result is in line with previously reported disadvantages associated with the liquid formulation: difficulty in handling the bottle, the large volume of a single dose, the unpleasant taste, and the infantilizing perception of the thick consistency and the relative need for a graduated syringe^18,21^.

When analyzing the level of satisfaction in relation to the frequency of alteration, PPS showed that the more the patients needed to manipulate the formulation (tablets or oral suspension), the more they were dissatisfied with the treatment. This effect was significant in tablet users (*p* < 0.0001) but not in oral suspension users, possibly due to the small size of the subgroup.

Together with general dissatisfaction, manipulation also leads to other relevant consequences, such as potential underdosing. A recent experimental study demonstrated that crushing riluzole tablets resulted in a mean percentage loss of powder of 6.27% and a mean percentage loss of active principle ingredient of 8.54%^23^. This is consistent with other experimental studies involving other active principles. In this body of evidence, crushing tablets showed a loss of active principle ingredients above the limit of FDA recommendations in case of manipulation (3%)^24,25^. This event can jeopardize the effectiveness of riluzole treatment and lead to early discontinuation or poor adherence, as shown in other studies^18–20,26,27^.

The difficulty in swallowing was also the reason for switching/interrupting treatment in 50% of PALS who switched/interrupted the therapy; as expected, dysphagic PALS viewed choking as a more serious risk than non dysphagic ones, possibly due to the fact that dysphagic patients had previously experienced a choking event. Both tablet and oral suspension users were least satisfied with the formulation in general, the ease of preparation, and self-administration.

PPS showed that the first crucial unmet need that should be addressed by a new formulation is an improvement in long-lasting manageability and swallowability providing a treatment that may facilitate a better continuity of riluzole administration and optimizing its efficacy. This would ultimately have a positive impact on the progression of the disease.

Since the late 1970s, various bioadhesive mucosal forms have been developed to overcome swallowing difficulties in children and geriatric patients, helping people who are afraid of choking^22^. The oral film is practical to use, it does not require water, there is no risk of choking, it is more accurately dosed compared to the liquid formulation or crushed tablets, and it presents fewer problems related to unpleasant taste^22,28^. An oral film formulation is a tiny layer enclosed in a pouch that can be stored at room temperature, opened by the PALS or caregiver, and placed on the tip of the tongue. The oral film formulation dissolves quickly without the need for a liquid or chewing and, once the film has dissolved completely, the released riluzole can be ingested^29^.

Besides identifying the unmet needs of PALS, PPS also assessed the levels of attractiveness of typical oral film characteristics: PALS preferred a formulation that did not require manipulation when administered and ease of self-administration. They also rated good oral-dissolving properties and no need for water as preferred attributes over the reduced risk of underdosing and a reduced metallic taste, regardless of the presence of dysphagia.

Overall, the attributes of a new oral film were positively perceived: PALS would prefer a formulation that avoids underdosing and preserves independence and self-administration for as long as possible. Therefore, the results of PPS may provide evidence for the usefulness of a new oral film formulation.

This research has some limitations that should be considered. (1) Due to the qualitative nature of the survey, the patients did not have the same experience with the formulation of the future treatments proposed, and this could have affected their answers. (2) Information regarding the cognitive status of the patients, as well as clinical evaluation of disease status, were not included. This is mainly due to the nature of this research which did not involve any clinician in the data collection but rather investigated the PALS’s perspective without any other conditioning. Moreover, the research did not aim at exploring the relation between the clinical disease status and the collected variables. The survey included some questions about ALS characteristics and diagnosis only with a descriptive intent. (3) The reduced number of patients limits the generalization of the results and does not allow for drawing firm conclusions but rather generates an interesting hypothesis that merits further investigation. (4) Due to the focus of the research on highly specific and original aspects of PALS preference, no validated scale could be used to evaluate peculiar aspects of QoL or treatment satisfaction. Given the interesting results of this research, we strongly support the need for real-world studies focusing on QoL and PALS preferences on treatments.

## Conclusions

To the best of our knowledge, this study is the first qualitative report of PALS preferences for different riluzole formulations. The findings suggest that PALS are not fully satisfied with current riluzole formulations and would welcome a new formulation that offers ease of administration and minimal impact on daily routine practice. Furthermore, they would appreciate using the same treatment during the course of the disease. An oral film technology could meet their needs, as an innovative way for PALS to take riluzole from the onset of the disease. Oral film ensures a convenient and precise dose that can be taken regardless of swallowing difficulties, and it could provide treatment continuity throughout the course of the disease. We encourage further development of a riluzole formulation that is suitable for the various stages of the disease, ensuring the correct daily dosing, without any need for manipulation needed, while enhancing the quality of life of people with ALS.

## Methods

### Study design and data source

The PPS was a survey carried out from June 13th, 2022 to July 25th, 2022 in four European countries (France, Germany, Italy, and Spain). The primary objective was to describe the unmet needs of current ALS treatment from a person’s perspective and express their compellingness for the characteristics of a new riluzole formulation. PALS were recruited in three different ways: by (1) disseminating the survey through local patient databases of ALS-treating physicians; (2) contacting ALS patient associations and (3) contacting ALS patients support groups on social media. No clinical institution was directly involved. The survey interviewed PALS or caregivers of PALS who were unable to speak or use a computer autonomously. The intention to participate in the interview was confirmed by a one-to-one telephone interview in order to assess inclusion criteria.

After recruitment, an online survey was conducted to assess treatment history, experiences and preferences and also included a QoL interview. The original survey written in English was translated into the respondent’s native language before being distributed.

The responses to the online survey were collected by “CONFIRMIT Horizon™”, a world-leading provider of advanced web-based data collection and reporting software that met the highest security and privacy standards. Informed consent about data protection was gained via the online survey/Confirmit system. Consent according to GDPR regulation was collected through the survey provider, no other ethical requirements were applicable. Data were made available for analysis in a de-identified research database. Any information participants reported is kept strictly confidential and anonymous. No personal identifying information or IP addresses were collected during the course of this research.

In addition to the survey, PPS included qualitative follow-up phone interviews with two patients or caregivers for each country. The PALS for the qualitative interview responded to the same selection criteria as for the survey. These 60-min guided interviews had several objectives: to validate the intelligibility of the survey for the participants; to assess the effort (and time) required to complete the survey and obtain further rationale behind the answers to the survey; to obtain a valid interpretation of the answers from perspectives of patients and caregivers.

The survey was for market research only and had no promotional intent, complying with all codes of the ABPI/MRS/BHBIA/ESOMAR/ICC/CCPA and Data Protection Act. All participants provided specific informed consent to participate in the research as well as to give the permission to collect and use their personal information, acknowledged their understanding of their rights to privacy, and to give consent to participate in the market research.

The survey protocol was reviewed and validated by Advarra’s Institutional Review Board (IRB) using the Department of Health and Human Services regulations found at 45 CFR 46.104(d)(2) and was determined as being exempt from IRB oversight, meaning the research was ethically designed. All methods were performed in accordance with the WMA Declaration of Helsinki.

The survey questionnaire is reported in Supplementary 2.

A Steering Committee—comprised of two clinicians with specific expertise in ALS and a patient representative of the European Organization for Professionals and Patients with ALS (EUpALS)—validated the key assumptions, rationale, and design of the study as well as the relevance and appropriateness of the questions. The Steering Committee was also involved in data analysis and interpretation of the aggregated data. results. The Steering Committee was blinded on the subjects' responses. The Steering Committee was composed of:

1. Albert C. Ludolph, Prof. MD, German Center for Neurodegenerative Diseases (DZNE), and Department of Neurology, University of Ulm, Ulm, Germany
2. Evy Reviers, European Organization for Professionals and Patients with ALS (EUpALS), Leuven, Belgium, ORCID: 0000-0002-6044-5234
3. Vincenzo Silani, Prof. MD., Department of Neuroscience & Laboratory of Neuroscience, IRCCS Istituto Auxologico Italiano, Milan, Italy, Dino Ferrari Center, Department of Pathophysiology and Transplantation, Università degli Studi di Milano, Milan ORCID: 0000-0002-7698-3854

Members of the Steering Committee are also co-authors of the article.

### Patient eligibility

Participants were PALS (or their caregivers) who met the following criteria: people diagnosed with ALS, whether bulbar, spinal, with mixed and unknown onset, at all stages; people on ALS treatments (riluzole or others), along with naïve patients. PALS involved in clinical trials could be included in the study. The study pre-specified a soft quota of PALS with several characteristics: presence/absence of dysphagia (65% vs. 35%, respectively); PALS treated with medication other than riluzole (10%); PALS with ALS symptoms for less than one year (10% of patients). Caregivers took part, only if the patients could not take the survey themselves. Caregivers were primary caregivers only, who were at least involved in healthcare and daily living care activities and were familiar with ALS treatment and care for severe PALS.

The survey excluded: adults unable to consent; people younger than 20 years of age; and caregivers not involved in the primary care of PALS.

### Statistical analysis

The statistical analyses were done on all respondents. In general, qualitative classification variables were descriptively summarized using absolute and relative frequencies, while mean, standard deviation, median, minimum, and maximum were used for continuous or ordinal variables. The Nonparametric Mann–Whitney U test was employed for statistical comparisons of outcomes expressed on ordinal scales (scores) across subgroups of particular clinical interest. All statistical analyses were performed using the procedures FREQ, MEAN, and NPAR1WAY of SAS software Version 9.4.

### Ethics approval and consent to participate

The survey protocol has been reviewed and validated by Advarra’s Institutional Review Board (IRB) using the Department of Health and Human Services regulations found at 45 CFR 46.104(d)(2) and was determined as being exempt from IRB oversight, meaning the research was ethically designed. All methods were performed in accordance with the WMA Declaration of Helsinki. All participant consented to the collection and use of their personal information, acknowledged their understanding of their rights to privacy, and consented to participate in the market research.

## Supplementary Information

Supplementary Information 1.

Supplementary Information 2.

## Data Availability

The data that support the findings of this study are available from Charles River Associates International, but restrictions apply to the availability of these data, which were used under license for the current study, and so are not publicly available. Data are however available from the authors upon reasonable request and with permission of Charles River Associates International. Please contact hgrandjean@crai.com for a request.
