# Author Correction: The preferences of people with amyotrophic lateral sclerosis on riluzole treatment in Europe

**DOI:** 10.1038/s41598-025-95009-7

**Published:** 2025-05-01

**Authors:** Albert C. Ludolph, Harish Grandjean, Evy Reviers, Valentina De Micheli, Cosetta Bianchi, Leonardo Cardosi, Hermann Russ, Vincenzo Silani

**Affiliations:** 1 Department of Neurology, German Center for Neurodegenerative Diseases (DZNE), University of Ulm, Ulm, Germany; 2 Charles River Associates International, Zürich, Switzerland; 3 European Organization for Professionals and Patients With ALS (EUpALS), Leuven, Belgium; 4 Business Integration Partners S.p.A, Milan, Italy; 5 Zambon Biotech SPA, Bresso, Milan, Italy; 6 Sirius Scientific Consulting AG, 8852 Altendorf, Switzerland; 7 Department of Neuroscience and Laboratory of Neuroscience, IRCCS Istituto Auxologico Italiano, Milan, Italy; 8 Department of Pathophysiology and Transplantation, Dino Ferrari Center, Università degli Studi di Milano, Milan, Italy

Correction to: *Scientific Reports* 10.1038/s41598-023-49424-3, published online 15 December 2023

“The original version of the Article contained errors.

There were discrepancies in the presentation and description of some findings due to incomplete revision of text, tables and figures.

In the Abstract,

“The dysphagic PALS were least satisfied with the riluzole tablet and oral suspension and with ease in self-administration; up to 68% of respondents postponed or missed the treatment due to swallowing difficulties and need of caregiver assistance. Overall, 51% of tablet and 53% of oral suspension users regularly crushed or mixed riluzole with beverages, respectively; PALS who always manipulated riluzole showed low satisfaction with the formulation and considered the risk of choking and pneumonia the most worrisome event.”

now reads:

“The dysphagic PALS were least satisfied with the riluzole tablet and oral suspension and with ease in self-administration; up to 46% of respondents postponed or missed the treatment due to swallowing difficulties and need of caregiver assistance. Overall, 51% of tablet and 50% of oral suspension users regularly crushed or manipulated riluzole, respectively; PALS who always manipulated riluzole showed low satisfaction with the formulation and considered the risk of choking and pneumonia the most worrisome event.”

In the Results section, “Mean Satisfaction with the current riluzole formulations”,

“Oral suspension users did not appreciate manageability (2.9/5), ease of self-administration (2.7/5) and, interestingly, the formulation itself (2.8/5) (Table 1)”

now reads:

“Oral suspension users did not appreciate manageability (2.9/5), ease of self-administration (2.7/5) and, interestingly, the formulation itself (2.9/5) (Table 1)”

In the Results section, “Manipulating riluzole oral suspension”,

“In particular, patients who regularly manipulate oral suspension showed low satisfaction with the formulation (2.0/5), with the easiness of self-administration, and with preparing the treatment before administration (2.4/5) as well as moderate satisfaction with package weight and size (3.3/5) (Table 3).”

now reads:

“In particular, patients who regularly manipulate oral suspension showed low satisfaction with the formulation (2.0/5), with the easiness of self-administration, and with preparing the treatment before administration (2.4/5 and 2.8/5) as well as moderate satisfaction with package weight and size (3.3/5) (Table 3).”

In the Results section, “Driving factors for choosing a different formulation”,

“The PALS also gave high scores for better self-administration (3.9/5), good oral-dissolving properties without tongue motility and no need for water or salivary stimulation (both 3.8/5). The survey results revealed mean scores above 3.0/5. also for lower risk of underdosing and lower risk of microbial contamination (both 3.7/5), followed by the reduction of metallic taste (3.6/5).”

now reads:

“The PALS also gave high scores for better self-administration (3.9/5), good oral-dissolving properties without tongue motility and no need for water or salivary stimulation (3.9/5 and 3.8/5 respectively). The survey results revealed mean scores above 3.0/5 also for lower risk of underdosing and lower risk of microbial contamination (3.8/5 and 3.7/5 respectively), followed by the reduction of metallic taste (3.7/5).”

In Table 4, “Desired characteristics of a riluzole formulation expressed by all participants surveyed and analyzed overall and per formulation used” dysphagic users were incorrectly referred to as non-dysphagic and vice versa. The incorrect and correct versions of Table 4 appear below.

Incorrect Table 4:

**Table:** 

Variable	Tablet user (n = 72)	Oral Suspension user (n = 32)	Dysphagic (n = 33)	Non dysphagic (n = 76)	All Patients (n = 109)
Driving factors for the choice new hypothetical product (Average)					
Mean ± SD	3,8 ± 1.0	3,9 ± 0,9	3,7 ± 0,9	4.0 ± 1.0	3,9 ± 1.0
Compellingness: It can dissolve in the mouth without the need to engage the tongue					
Mean ± SD	3,9 ± 1,4	4,00 ± 1,50	3,8 ± 1,4	4,00 ± 1,4	3,9 ± 1,4
Compellingness: No need for water and no salivary stimulant					
Mean ± SD	3,7 ± 1,5	4.0 ± 1,4	3,5 ± 1,5	4.0 ± 1,5	3,8 ± 1,5
Compellingness: Reduced risk of contamination* compared with available treatments					
Mean ± SD	3,6 ± 1,4	3,7 ± 1,1	3,3 ± 1,3	3,8 ± 1,3	3,7 ± 1,3
Compellingness: Reduced risk of underdosing** compared with available treatments					
Mean ± SD	3,7 ± 1,4	4,00 ± 1, 2	3,4 ± 1,2	4.0 ± 1,3	3,8 ± 1,3
Compellingness: Reduced metallic taste compared with available treatments					
Mean ± SD	3,6 ± 1,4	4,00 ± 1,2	3,3 ± 1,5	3,9 ± 1,2	3,7 ± 1,3
Compellingness: Potential self-administration and independence					
Mean ± SD	3,9 ± 1,4	3, 9 ± 1,50	4,0 ± 1,24	3,9 ± 1,5	3,9 ± 1,4
Compellingness: Intuitive and easy use without the need for extensive instruction					
Mean ± SD	4,3 ± 1.0	4,1 ± 1,4	4,4 ± 0,90	4,2 ± 1,2	4,3 ± 1,10
Compellingness: Convenient and portable packaging					
Mean ± SD	4,00 ± 1,2	3,8 ± 1,1	3,8 ± 1,4	4,0 ± 1,1	4.0 ± 1,2

Correct Table 4:

**Table:** 

Variable	Tablet user (n = 72)	Oral suspension user (n = 32)	Dysphagic (n = 33)	Non dysphagic (n = 76)	All patients (n = 109)
Driving factors for the choice new hypothetical product (average)					
Mean ± SD	3,8 ± 1.0	3,9 ± 0,9	3,7 ± 0,9	4.0 ± 1.0	3,9 ± 1.0
Compellingness: It can dissolve in the mouth without the need to engage the tongue					
Mean ± SD	3,9 ± 1,4	4,00 ± 1,50	3,8 ± 1,4	4,00 ± 1,4	3,9 ± 1,4
Compellingness: No need for water and no salivary stimulant					
Mean ± SD	3,7 ± 1,5	4.0 ± 1,4	3,5 ± 1,5	4.0 ± 1,5	3,8 ± 1,5
Compellingness: Reduced risk of contamination* compared with available treatments					
Mean ± SD	3,6 ± 1,4	3,7 ± 1,1	3,3 ± 1,3	3,8 ± 1,3	3,7 ± 1,3
Compellingness: Reduced risk of underdosing** compared with available treatments					
Mean ± SD	3,7 ± 1,4	4.00 ± 1,2	3,4 ± 1,2	4.0 ± 1,3	3,8 ± 1,3
Compellingness: Reduced metallic taste compared with available treatments					
Mean ± SD	3,6 ± 1,4	4,00 ± 1,2	3,3 ± 1,5	3,9 ± 1,2	3,7 ± 1,3
Compellingness: Potential self-administration and independence					
Mean ± SD	3,9 ± 1,4	3, 9 ± 1,50	4,0 ± 1,24	3,9 ± 1,5	3,9 ± 1,4
Compellingness: Intuitive and easy use without the need for extensive instruction					
Mean ± SD	4,3 ± 1.0	4,1 ± 1,4	4,4 ± 0,90	4,2 ± 1,2	4,3 ± 1,10
Compellingness: Convenient and portable packaging					
Mean ± SD	4,00 ± 1,2	3,8 ± 1,1	3,8 ± 1,4	4,0 ± 1,1	4.0 ± 1,2

In the Discussion section,

“This study also showed that, due to swallowing difficulties or lack of independence, more than 68% of PALS postpone or miss a treatment; therefore, dysphagia onset may also have a significant impact on treatment adherence.”

now reads:

“This study also showed that, due to swallowing difficulties or lack of independence, more than 46% of PALS postpone or miss a treatment; therefore, dysphagia onset may also have a significant impact on treatment adherence.”

Additionally,

“More than half (56%) of the tablet users crushed tablets to facilitate swallowing.”

now reads:

“51% of the tablet users crushed tablets to facilitate swallowing.”

Furthermore,

“The difficulty in swallowing was also the reason for switching/interrupting treatment in 40% of PALS who switched/interrupted the therapy; as expected, dysphagic PALS viewed choking as a more serious risk than non dysphagic ones, possibly due to the fact that dysphagic patients had previously experienced a choking event.”

now reads:

“The difficulty in swallowing was also the reason for switching/interrupting treatment in 50% of PALS who switched/interrupted the therapy; as expected, dysphagic PALS viewed choking as a more serious risk than non dysphagic ones, possibly due to the fact that dysphagic patients had previously experienced a choking event.”

In the Supplementary Information 2 file, Supplementary Table 2, the percentage of patients who switched or interrupted treatment has been corrected. Supplementary Figure 1 in the same file has been replaced by a new figure showing the correct data regarding the tablet manipulation practices of the patients. The original Supplementary Information 2 file is linked to this correction notice.

The original Article has been corrected.

## Supplementary Information

Supplementary Information.

