# Supplementary material for: The preferences of people with amyotrophic lateral sclerosis on riluzole treatment in Europe
Source: Sci Rep. 2023 Dec 15;13:22497. doi: 10.1038/s41598-023-49424-3 (PMC10728064; doi:10.1038/s41598-023-49424-3)
Supplement: Supplementary file 1 — Supplementary Information 1. [file 41598_2023_49424_MOESM1_ESM.pdf]

Zambon – ROF Patient Preference Study

ALS Patient and Caregiver Questionnaire

Q1 2022

Survey guide v5.4

1st of June 2022

CONFIDENTIAL

Prepared by
Charles River Associates

FOR MARKET RESEARCH PURPOSES ONLY

SECTION S - Screener [DO NOT SHOW]

QUOTA			
Country	# of Patients	# of Caregivers	Total Patients/Caregivers
DE	15	15	30
FR	15	15	30
ES	15	15	30
IT	15	15	30
TOTAL	~60	~60	120

QUOTA TABLE (DO NOT SHOW)

RECRUITER NOTE: PLEASE KEEP A RECORD OF ALL SCREEN-OUTS AND FEEDBACK TO THE TEAM

SOFT QUOTA			
COUNTRY	# of patient (or caregiver caring for patients) WITHOUT dysphagia	# of patient (or caregiver caring for patients) WITH dysphagia	Total Patients/Caregivers
DE	10	20	30
FR	10	20	30
ES	10	20	30
IT	10	20	30
TOTAL	~40	~80	120

SOFT QUOTA S90	
RILUZOLE TREATMENT NAÏVE PATIENTS	
	~10% OF TOTAL RESPONDENT MAXIMUM
Total	~12

SOFT QUOTA S30	
EARLY PATIENTS	
	~10% OF TOTAL RESPONDENT MAXIMUM
Total	~12

Screener

INTRO TEXT

Thank you for taking the time to participate in this survey for Charles River Associates. We're conducting independent market research on behalf of a pharmaceutical company, regarding treatment preferences relating to Amyotrophic Lateral Sclerosis (ALS).

The questionnaire is expected to take approximately 30 minutes, and if you qualify, you will be remunerated as specified in the study invitation.

This survey is for market research only and has no promotional intent. It complies with all codes of the ABPI/MRS/BHBIA/ESOMAR/ICC/CCPA and Data Protection Act. Your responses will be recorded and provided to the sponsor but with no personally identifying information, so you will remain anonymous.

Charles River Associates (CRA) takes its data protection and privacy responsibilities seriously. This short privacy notice provides key information about the collection and use of your personal information during the Project. More detailed information about CRA's privacy practices, and about your rights, can be found in our full privacy statement at http://www.crai.com/privacy.

What personal information do we collect and how is it collected?

We collect personal information from you when you apply to take part in the Project, and during the course of the survey process. This information is likely to include:

- Any personal information which you volunteer during the survey

What do we use your information for?

Your personal information is collected for the primary purpose of carrying out research for the benefit of the Sponsor. We collect information about you throughout the survey process, and compile this into a report which we provide to the Sponsor. Except where we collect information about your health, race or ethnic origin (and you provide your explicit consent for us to do so), our processing of your personal information is based on the legitimate interest which we and the Sponsor have in generating, through research, intelligence about consumer habits and preferences which the Sponsor can use to develop more relevant products, and/or materials. Our legitimate interest is conditional on respecting your privacy rights, and you have a right to object to this processing at any time.

We may also be required to collect and retain certain personal information to comply with legal requirements relevant to the life sciences market research industry, and we retain the right to process your personal information if it is relevant to us establishing, exercising or defending our legal rights.

Your personal information may be sent outside of the European Economic Area and may also be shared with our third-party service providers, regulators, courts and government or law enforcement bodies, as described in our full Privacy Statement.

The information collected in the survey may also be collated and used in a peer-reviewed publication and will conform to the standard of ethical behaviour.

Your rights to Privacy

You have certain rights which you can exercise in relation to our use of your personal information - please find out more about these by reviewing our full Privacy Statement found here: http://www.crai.com/privacy.

Contact us

If you have any concerns or questions, you should contact: PrivacyMatters@crai.com.

Please indicate your acknowledgement by checking your consent to each item below to continue:

Code	**GDPR1**	**Select all that apply**	
1	I consent to my collection of personal information mentioned above	☐	**[PROCEED ONLY IF ALL SELECTED]**
2	I consent to the use of my personal information as mentioned above	☐	
3	I understand my rights to privacy as mentioned above	☐	

[DE ONLY] This project will comply with all applicable laws in Germany regarding the protection of your personal data and the guidelines of the European Pharmaceutical Marketing Research Association (EphMRA), the European Society for Opinion and Marketing Research (ESOMAR) and the Working Group of German Market and Social Research Institutes e.V. (ADM). This means in particular:

- This study is not for advertising purposes. Your answers will only be used for market research purposes.
- Your answers will be kept confidential and will not be used for other purposes or shared with third parties.
- Of course, you always have the right to refuse individual answers or to cancel the survey.

[GERMAN TRANSLATION OF ABOVE TEXT] Dieses Projekt erfüllt alle in Deutschland geltende Gesetze hinsichtlich dem Schutz Ihrer persönlichen Daten sowie die Richtlinien der European Pharmaceutical Marketing Research Association (EphMRA), der European Society for Opinion and Marketing Research (ESOMAR), und des Arbeitskreis Deutscher Markt- und Sozialforschungsinstitute e.V. Dies bedeutet insbesondere:

- Diese Studie dient nicht zu Werbezwecken. Ihre Antworten werden nur für Marktforschungszwecke verwendet.
- Ihre Antworten werden vertraulich behandelt und nicht für andere Zwecke verwendet oder an Dritte weitergegeben.
- Natürlich können Sie jederzeit einzelne Antworten ablehnen oder die Umfrage abzubrechen.

[ALL] We are required to pass on to our client details of adverse events and / or product complaints that are mentioned during the collection of market research. Although what you say will be treated in confidence, should you raise during the survey an adverse event and/ or product complaint, we will need to report this to the client even if it has already been reported by you directly to the company or the regulatory authorities. In such a situation, you will be asked whether you consent to have your identity revealed to the client. Everything else you say during the survey will continue to remain confidential, and you will still have the option to remain anonymous if you so wish. Are you willing to participate with the survey on this basis?

1	Yes, I would like to proceed	**[CONTINUE]**
2	No, I do not want to proceed	**[TERMINATE IMMEDIATELY]**

[DE ONLY] Pharmaunternehmen sind gesetzlich dazu verpflichtet, Meldungen unerwünschter Ereignisse und anderer meldepflichtiger Ereignisse zu erfassen und an die Zulassungsbehörden zu melden. Sollten Sie während der Befragung ein unerwünschtes Ereignis und / oder ein anderes meldepflichtiges Ereignis bei einem bestimmten Patienten ansprechen, so müssen wir dieses an das pharmazeutische Unternehmen weiterleiten. Auch wenn Sie selbst schon direkt beim Unternehmen oder bei der Aufsichtsbehörde das unerwünschte Ereignis und / oder andere meldepflichtige Ereignisse gemeldet haben sollten, sind wir verpflichtet, den Fall zu erfassen und zu melden.

Die Meldung erfolgt gemäß der berufsständischen „Richtlinie für Studien im Gesundheitswesen zu Zwecken der Markt- und Sozialforschung" des Arbeitskreises Deutscher Markt- und Sozialforschungsinstitute e.V. (ADM) anonym.

Sind Sie damit einverstanden, unter diesen Voraussetzungen das Interview durchzuführen?

1	Ich würde gerne teilnehmen	**[CONTINUE]**
2	Ich möchte nicht teilnehmen und die Befragung an dieser Stelle beenden	**[TERMINATE IMMEDIATELY]**

Stimmen Sie zu, ggf. nochmals kontaktiert zu werden, sofern die Arzneimittelsicherheit des beauftragenden Pharmaunternehmens weitere Informationen zu einem eventuellen Meldefall benötigen sollte? Auch in diesem Fall bliebe Ihre vollständige Anonymität entsprechend berufsständischer Richtlinien garantiert; alle Ihre Auskünfte würden auch in diesem Fall anonym erfolgen. Zur Erfüllung regulatorischer Anforderungen würden Ihre Kontaktdaten beim Vorkommen meldepflichtiger Ereignisse für eventuelle Rückfragen der Arzneimittelsicherheit maximal ein Jahr lang durch die Marktforschungsagentur aufbewahrt.

1	Ja	**[CONTINUE]**
2	Nein	**[TERMINATE IMMEDIATELY]**

[IF TERMINATED] Unfortunately, you do not qualify to participate in our research. We appreciate the time that you took to answer these questions. We will notify you if other opportunities for research arise. Thank you again.

[IF QUALIFIED]: “Thank you for your time and interest in this research. Based on your responses, you have qualified to proceed to the main survey. This survey will take approximately 30-minutes of your time. Please proceed to the next screen to begin.”

S10. Which country are you based in?

Code	[RANDOMIZE, ANCHOR OTHER TO BOTTOM]	Select one
1	Germany	□ [PROCEED]
2	Italy	□ [PROCEED]
3	Spain	□ [PROCEED]
4	France	□ [PROCEED]
99	Other, please specify [OPEN FREE TEXT BOX]	□ [TERMINATE]

S20. Have you or anyone in your household been diagnosed with Amyotrophic Lateral Sclerosis?

Code		I have been diagnosed with ALS [CATEGORIZE AS PATIENT]	Someone in my household has been diagnosed with ALS [CATEGORIZE AS CAREGIVER]
1	Yes	□ [PROCEED]	□ [PROCEED]
2	No	□ [TERMINATE]	□ [TERMINATE]

S30. When did you/the person with ALS you care for first experience ALS related symptoms?

Code		Select one
1	Less than 1 year	□ [PROCEED] [COUNT AS "EARLY" SOFT QUOTA]
2	1 to 3 years	□ [PROCEED]
3	3 to 5 years	□ [PROCEED]
4	More than 5 years	□ [PROCEED]

S40. Which of the statements below best describes your ALS (or the person with ALS you care for)?

Code	[RANDOMIZE, KEEP I DON'T KNOW TO BOTTOW]	Select one

1	Spinal onset (typically, the first symptoms appear in the arms or legs)	□ [PROCEED]
2	Bulbar onset (typically, the first symptoms appear in the head, with speech or swallowing difficulties)	□ [PROCEED]
3	Mixed onset	□ [PROCEED]
99	I don't know	□ [PROCEED]

S50. [ASK CAREGIVER ONLY] Are you the caregiver (eg. parent or guardian) for someone who has been diagnosed with Amyotrophic Lateral Sclerosis (ALS)?

Code	[RANDOMIZE]	Select one
1	Yes, I am the primary caregiver of someone with ALS	□ [PROCEED]
2	No. I am not the primary caregiver but am involved in some healthcare and daily living care activities. I am familiar with the treatment taking habits of someone with ALS	□[PROCEED]
3	No. I am not the primary caregiver but am involved in other ways such as financial support, social support...etc.	□ [TERMINATE]

S60. [IF S50_R1] As the caregiver of a person living with ALS, do you routinely do the following?

Code	[RANDOMIZE, ANCHOR NONE TO BOTTOM]	Multi select	
1	I take them to medical appointments	□	[PROCEED IF AT LEAST S60_R4 OR S60_R5 ARE SELECTED]
2	I discuss treatment options with healthcare providers	□	
3	I make treatment decisions in conjunction with healthcare providers	□	
4	I help them manage their needs related to daily living (bathing, feeding)	□	
5	I help them with the administration of their treatment	□	

99	None of the above	□ [TERMINATE]

S70. How would you describe the feeding needs for you or the person with ALS you are caring for?

Code	[RANDOMIZE, KEEP I DON'T KNOW TO BOTTOM]	Select one
1	Normal alimentary habits	□ [PROCEED]<br>Classify as "Without dysphagia"
2	Problems with feeding, eventual choking	□ [PROCEED]<br>Classify as "With dysphagia"
3	Changes in the food consistency (provoked by difficulty in swallowing)	□ [PROCEED]<br>Classify as "With dysphagia"
4	Necessity of supplement tube for feeding	□ [PROCEED]<br>Classify as "With dysphagia"
5	Complete dependence to feeding tube	□ [PROCEED]<br>Classify as "With dysphagia"
99	I do not know	□ [TERMINATE]

S80. Which of the following statements best describe the functionality you have given your ALS (or for the person with ALS you care for)? Please select all applicable responses.

Code	[RANDOMIZE]	Select one per category
**Speech**		**Single select**
1	I am able to communicate intelligibly	□ [PROCEED WITH PATIENT ONLY]<br>[TERMINATE CAREGIVER ONLY]
2	I have a loss of useful speech	□ [PROCEED]
**Typewriting**		**Single select**
5	I am able to type on a keyboard /computer	□ [PROCEED WITH PATIENT ONLY]<br>[TERMINATE CAREGIVER ONLY]
6	I am not able to write	□ [PROCEED]
**Alimentary habits**		**Single select**

7	I do not have a gastrostomy and am able to feed myself without help	□ [PROCEED WITH PATIENT ONLY]<br>[TERMINATE CAREGIVER ONLY]
8	I do not have a gastrostomy but I need help to feed myself	□ [PROCEED]
9	I have a gastrostomy and need minimal assistance	□ [PROCEED]
10	I have a gastrostomy and I need significant assistance	□ [PROCEED]
**Dressing and hygiene**		**Single select**
11	I am independent in my dressing and hygiene	□ [PROCEED WITH PATIENT ONLY]<br>[TERMINATE CAREGIVER ONLY]
12	I need significant assistance for self-care	□ [PROCEED]

S90. Which of the following treatment options have you ever been prescribed for treating Amyotrophic Lateral Sclerosis (ALS)? Please select all applicable responses.

Code	[RANDOMIZE, ANCHOR OTHER TO BOTTOM]	Multi select
1	Riluzole generic or Rilutek (tablet formulation)	□ [PROCEED]
2	Teglutik (liquid formulation)	□ [PROCEED]
3	Edaravone (intravenous)	□ [PROCEED] [CLASSIFY AS TX NAÏVE; SEE SOFT QUOTA]
4	No treatment	□ [PROCEED] [CLASSIFY AS TX NAÏVE; SEE SOFT QUOTA] [IF SELECTED, DO NOT ALLOW OTHER SELECTION]
99	Other, please specify [OPEN FREE TEXT BOX]	□ [PROCEED] [CLASSIFY AS TX NAÏVE; SEE SOFT QUOTA]

S100. Are you currently involved, or have you participated up to two months ago in an Amyotrophic Lateral Sclerosis (ALS) clinical trial?

Code	[RANDOMIZE, KEEP NONE AT THE BOTTOM]	Multi select
1	Yes	□ [PROCEED]

2	No	□ **[PROCEED]**
3	**[IF S100_R1]** If you are aware, please specify the company: **[OPEN FREE TEXT BOX]**	□ **[PROCEED]**

S110. **[IF S100_R1 ~~NOT~~ SELECTED]** For the clinical trial you have participated in, which of the options below best describe the type of treatment tested?

Code	**[RANDOMIZE, KEEP OTHER TO BOTTOM]**	**Select one**
1	Pharmaceutical treatment	□ **[PROCEED]**
2	Non-pharmaceutical treatment	□ **[PROCEED]**
3	I don't know	□ **[PROCEED]**

[RETAIN ALL SCREENER INFO AND PROVIDE TO CRA]

THANK AND TERMINATE AT THIS POINT AND NOT BEFORE

[IF TERMINATED] Unfortunately, you do not qualify to participate in our research. We appreciate the time that you took to answer these questions. We will notify you if other opportunities for research arise. Thank you again.

[IF QUALIFIED]: "Thank you for your time and interest in this research. Based on your responses, you have qualified to proceed to the main survey. This survey will take approximately 30 minutes of your time. Please proceed to the next screen to begin."

Thank you for agreeing to participate in this Amyotrophic Lateral Sclerosis related project ("Project"). Charles River Associates ("CRA" or "we", "us") takes its data protection and privacy responsibilities seriously. This short privacy notice provides key information about the collection and use of your personal information during the Project. More detailed information about CRA's privacy practices, and about your rights, can be found in our full privacy statement at http://www.crai.com/privacy.

This survey is for market research only and has no promotional intent. It complies with all codes of the ABPI/MRS/BHBIA/ESOMAR/ICC/CCPA and Data Protection Act. Your responses will be recorded and provided to the sponsor but with no personally identifying information, so you will remain anonymous.

What personal information do we collect and how is it collected?

We collect personal information from you when you apply to take part in the Project, and during the course of the survey process. This information is likely to include:

- Any personal information which you volunteer during the survey

What do we use your information for?

Your personal information is collected for the primary purpose of carrying out research for the benefit of the Sponsor. We collect information about you throughout the survey process, and compile this into a report which we provide to the Sponsor. Except where we collect information about your health, race or ethnic origin (and you provide your explicit consent for us to do so), our processing of your personal information is based on the legitimate interest which we and the Sponsor have in generating, through research, intelligence about consumer habits and preferences which the Sponsor can use to develop more relevant products, and/or materials. Our legitimate interest is conditional on respecting your privacy rights, and you have a right to object to this processing at any time.

We may also be required to collect and retain certain personal information to comply with legal requirements relevant to the life sciences market research industry, and we retain the right to process your personal information if it is relevant to us establishing, exercising or defending our legal rights.

Your personal information may be sent outside of the European Economic Area and may also be shared with our third-party service providers, regulators, courts and government or law enforcement bodies, as described in our full Privacy Statement.

Your rights to Privacy

You have certain rights which you can exercise in relation to our use of your personal information - please find out more about these by reviewing our full Privacy Statement found here: http://www.crai.com/privacy.

Contact us

If you have any concerns or questions, you should contact: PrivacyMatters@crai.com.

You are about to participate in a market research survey. We are now being asked to pass on to our client details of adverse events and / or product complaints that are raised during the course of market research interviews. Your responses will, of course, be treated in confidence, should you raise an adverse event and / or product complaint, we will need to report this, even if it has already been reported by you directly to the company or the regulatory authorities using the MHRA's 'Yellow Card' system. In such a situation you can decide whether or not your contact details are to be passed on to the pharmacovigilance team. You have the option to waive the confidentiality given to you under the market research codes of conduct specifically in relation to that adverse event

and / or product complaint. Everything else you contribute during the course of the interview will continue to remain confidential.

Are you happy to participate in the interview on this basis?

Code		Select one
1	I would like to proceed and give permission for my contact details to be passed on to the Drug Safety department of the company if an adverse event is mentioned by me during the survey	**[CONTINUE]**
2	I would like to proceed and protect my anonymity	**[CONTINUE]**
3	I don't want to proceed and would like to end the interview here	**[THANK & TERMINATE]**

[END OF SCREENER]

NOTES BELOW NOT SEEN BY RESPONDENTS

Section S:

- Country
- Time since symptom onset
- Onset type
- Level of care from caregiver
- Level of dysphagia
- Level of functionality
- Treatment

Section A (1 min): Patient Background Information

- Age

Section B (12-13 min): Patient history

- Time of first symptoms onset
- Time of diagnosis
- Treatment history
- Tablet practice
- Teglutik practice
- Future treatment practices
- Rating of treatment attributes
- Treatment satisfaction
- ROF attributes rating
- Compliance
- Reasons to change treatment
- Interaction with HCPs

Section C (7 min): Discrete Choice Experiment Without ROF

- DCE

Section D (7 min): Discrete Choice Experiment With ROF

- DCE

Section E (3 min): Patient reported outcome & QoL

- QoL rating

SECTION A: PATIENT BACKGROUND INFORMATION – [DO NOT SHOW SECTION HEADER]

[SHOW FOR CAREGIVERS ONLY] For the purpose of this survey, please answer the following questions from the point of view of the person with ALS you are caring for, unless specified otherwise.

A20. [ASK PATIENTS ONLY] Could you please indicate in which age category you fall? [ASK CAREGIVERS ONLY] Could you please indicate in which age category the person with ALS you care for falls in?

Code	Age range	Select one
1	Less than 20 years	o
2	20-29 years	o
3	30-39 years	o
4	40-49 years	o
5	50-59 years	o
6	60-69years	o
7	70+ years	o
98	I prefer not to answer	o

SECTION B: PATIENT HISTORY – [DO NOT SHOW SECTION HEADER]

B30. [ASK PATIENTS ONLY] Please indicate if you are currently taking an ALS treatment. [ASK CAREGIVERS ONLY] Please indicate if the person with ALS you care for is currently taking an ALS treatment.

Code		Select one
1	Yes, I am currently taking an ALS specific treatment	□
2	No, I am currently not taking any ALS specific treatment	□

B50. [DO NOT ASK IF S90R4 SELECTED] [ASK PATIENTS ONLY] Which prescription treatment have you taken previously for your ALS? [ONLY IF S90R1+S90R2>1] You indicated that you took multiple ALS treatment. Please select all ALS treatments you have ever taken.
[DO NOT ASK IF S90R4 SELECTED] [ASK CAREGIVERS ONLY] Which prescription treatment has the person with ALS you care for taken previously for their ALS? [ONLY IF S90R1+S90R2>1] You indicated that the person with ALS you care for took multiple ALS treatment. Please select all ALS treatments they have ever taken.

Code	**Previously taken medication** [RANDOMIZE: KEEP OTHER TO BOTTOM]	**Medication taken 1st** Select one	**Medication taken 2nd** Select one	[SHOW ONLY IF B50 IS 3 ROWS] **Medication taken 3rd** Select one

1	[ONLY IF S90R1] Riluzole generic or Rilutek (tablet formulation)	☐	☐	☐
2	[ONLY IF S90R2] Teglutik (liquid formulation)	☐	☐	☐
98	[ONLY IF S90R4] No treatment		☐	
99	Other, please specify:_____ [ENABLE FREE TEXT]	☐	☐	☐

B61. [~~ONLY~~ IF S90R1+S90R2>1 OR B30R2 OR S90R4 NOT SELECTED] You indicated that you took multiple ALS treatments and/or that you interrupted your treatment. Please select the reasons that are applicable.

Code	**Treatment switch** [RANDOMIZE, KEEP OTHER TO BOTTOM]	**Multiselect**
1	I did not like the taste of the medicine	☐
2	I did not like the feeling in the mouth / mouth numbness	☐
3	I had difficulties to swallow the tablet formulation	☐
4	I had difficulties to swallow the oral suspension / thick liquid	☐
5	I did not like to bring bottles and syringes when travelling	☐
6	I did not like to crush the tablets	☐
7	My doctor decided to change the treatment	☐
99	Other, please specify:_____ [ENABLE FREE TEXT]	☐

B62. [ONLY IF S90R4 NOT SELECTED] Please indicate how easy to take you find the treatment(s) you have been prescribed. *Please indicate your response on a scale from 1 to 5, where 1 is "Not at all easy" and 5 is "Very easy".*

Code	**Convenience**	**Slide bar** [SLIDE BAR 1 = NOT AT ALL EASY - 5 = VERY EASY]
1	[ONLY IF S90R1] Riluzole or Rilutek tablets	[SLIDE BAR 1-5]
2	[ONLY IF S90R2] Teglutik (liquid formulation)	[SLIDE BAR 1-5]

B70. **[~~ONLY~~ IF B50R1 OR S90R4 NOT SELECTED]** We would like now to understand how you use the riluzole/Rilutek tablets. Please indicated below the options that best reflect your practice.

Code	**Tablet practice**	**Single select**
1	I currently take entire tablets without crushing them	☐
2	I have never crushed the tablet to facilitate swallowing	☐
3	I sometimes crush the tablet to reduce the size and facilitate swallowing	☐
4	I regularly/always crush the tablet to reduce the size and facilitate swallowing	☐
99	Other, please specify:_____ **[ENABLE FREE TEXT]**	☐

B71. **[IF B70R3 OR B70R4 OR S90R4 NOT SELECTED]** As you mentioned crushing the tablets, we would like now to understand how you do it. Please indicated below the options that best reflect your practice.

Code	**Tablet crushing practice**	**Multiselect**
1	I crush the tablet myself	☐
2	I ask my caregiver to crush the tablet	☐
3	I mix the crushed tablet with food	☐
4	I mix the crushed tablet with a liquid	☐
5	I crush the tablet directly on a table	☐
6	I crush the tablet on a piece of paper	☐
7	I crush the tablet on a piece of cloth	☐
8	I crush the tablet with a mortar and pestle	☐
9	I see residue of crushed tablet powder left that is not ingested	☐
10	I make sure that all the crushed tablet is taken, without leaving any leftover	☐
99	Other, please specify:_____ **[ENABLE FREE TEXT]**	☐

B80. **[IF B50R1 OR S90R4 NOT SELECTED] [ASK PATIENTS ONLY]** Please think about the future and indicate which statements below are most likely to reflect your practice.
[ASK CAREGIVERS ONLY] Please think about the future and indicate which statements below are most likely to reflect the practice of the person with ALS you care for.

Code	Future tablet practice	Multiselect
1	In the future, I intend to crush the riluzole tablet, if I can swallow it easier, after asking my doctor	☐
2	In the future, I intend to crush the riluzole tablet, if I can swallow it easier, without asking my doctor	☐
3	In the future, I intend to switch treatment to the liquid formulation	☐
4	In the future, I intend to stop treatment	☐
5	In the future, I intend to keep taking the treatment, as I do now	☐
99	Other, please specify:_____ **[ENABLE FREE TEXT]**	☐

B90. [IF B50R2 OR S90R4 NOT SELECTED] I would now like to understand how you use Teglutik. Please indicated below the options that best reflect your practice.

Code	Liquid formulation practice	Single select
1	I currently take Teglutik without altering its form	☐
2	I have never altered Teglutik to facilitate swallowing	☐
3	I sometimes alter the form of Teglutik to facilitate swallowing, such as mixing with other liquids	☐
4	I regularly alter the form of Teglutik to facilitate swallowing, such as mixing with other liquids	☐
99	Other, please specify:_____ **[ENABLE FREE TEXT]**	☐

B91. [IF B50R2 OR S90R4 NOT SELECTED] I would like to understand the different ways you use Teglutik. Please indicated below the options that best reflect your practice.

Code	Liquid formulation	Multiselect
1	I mix Teglutik with foods	☐
2	I mix Teglutik with liquids	☐
3	I take Teglutik through the feeding tube	☐
4	I use a syringe to administer the liquid orally	☐
5	I take Teglutik with a spoon	☐
99	Other, please specify:_____ **[ENABLE FREE TEXT]**	☐

B92. [IF B50R2 OR S90R4 NOT SELECTED] Please indicate for each statement below how they relate to your experience. *Please use a scale from 1 to 5, where 1 is "Not at all" and 5 is "Very much".*

Code	**Liquid formulation**	**Rate from 1 to 5 [SLIDE BAR 1 = NOT AT ALL - 5 = VERY MUCH]**
1	The big volume of Teglutik is bothersome	
2	The big volume of Teglutik is worrisome	
3	The big volume of Teglutik is dangerous	
4	Non-crushed pills are safer than the thick liquid of Teglutik	
5	Crushed pills are safer than the thick liquid of Teglutik	

B100. [ONLY IF B50R2] [ASK PATIENTS ONLY] Please think about the future and indicate which statements below are most likely to reflect your practice.
[ASK CAREGIVERS ONLY] Please think about the future and indicate which statements below are most likely to reflect the practice of the person with ALS you care for.

Code	**Future Teglutik practice**	**Multiselect**
1	In the future, I intend to mix Teglutik with food/liquid, if I can swallow it easier, after asking my doctor	□
2	In the future, I intend to mix Teglutik with food/liquid, if I can swallow it easier, without asking my doctor	□
3	In the future, I intend to switch treatment to the tablet formulation	□
4	In the future, I intend to stop treatment	□
5	In the future, I intend to keep taking the treatment, as I do now	□
99	Other, please specify:____ **[ENABLE FREE TEXT]**	□

[ONLY IF S90R4 NOT SELECTED] [SHOW B101 & B102 ON THE SAME PAGE]

B101. [ONLY IF S90R4 NOT SELECTED] [ASK PATIENTS ONLY] Many times, there is a lot going on in life and it can be difficult to take treatments as indicated. To understand your treatment taking habits, please indicate below which statements best apply to your experience.
[ONLY IF S90R4 NOT SELECTED] [ASK CAREGIVERS ONLY] Many times, there is a lot going on in life and it can be difficult to take treatments as indicated. To understand the treatment taking habits of the person with ALS you care for, please indicate below which statements best apply to their experience.

Code	Treatment taking habits	[NUMERICAL ENTRY; RANGE FROM 0 TO 100]
1	What % of the time do you take your treatment as planned, in a typical week?	___% of time
2	What % of time do you shift the time of taking treatment but still take your daily dose, in a typical week?	___% of time
3	[IF B101R2 >0%] How often does the postponing of taking the treatment lead to missing the daily treatment, in a typical week?	___% of time

B102. [ONLY IF S90R4 NOT SELECTED] [DO NOT SHOW IF B101R1 = 100] Please indicate for each of the statements below how often you postpone or miss a treatment. Please indicate you answers in for a **typical week.**

Code	**Reasons to skip treatments**	**Postponing a treatment in a week** [NUMERICAL ENTRY; RANGE FROM 0 TO 100]	**Missing a treatment in a week** [NUMERICAL ENTRY; RANGE FROM 0 TO 100]
1	Difficulty swallowing on that day	___% of time	___% of time
2	Difficulty to take the treatment myself	___% of time	___% of time
3	Need for a caregiver to assist	___% of time	___% of time
99	Other, please specify:______ [ENABLE FREE TEXT]	___% of time	___% of time
		[FORCE SUM TO 100%]	[FORCE SUM TO 100%]

B110. [ASK PATIENTS ONLY] Please select and rate the top 3 factors which are most important to you when choosing an ALS medication?
[ASK CAREGIVER ONLY] Please select and rate the top 3 factors which are most important to the person you care for when choosing an ALS medication?

Code	**Attribute preferences** [RANDOMIZE]	**Multiselect** [RATE SELECTION FROM 1 TO 3; LIMIT TO 3; DO NOT ALLOW TO PROCEED IF <3 OR >3 SELECTED]

1	The way in which I take a medication (as a pill or a liquid)	☐
2	Low risk of experiencing mild to moderate side effects, such as displeasing taste or numbing of the mouth	☐
3	Low to no risk of choking when ingesting the medication	☐
4	Low to no risk of developing severe gastrointestinal undesirable side effect	☐
5	Fits to my lifestyle	☐
6	My doctor recommended it	☐
7	Ability to self-administer	☐
99	Other, please specify:______ **[ENABLE FREE TEXT]**	☐

B120. [**IF S90R4 NOT SELECTED**] **[ASK PATIENTS ONLY]** How would you rate your satisfaction with the aspects of your current medication for ALS, listed below?
[**IF S90R4 NOT SELECTED**] **[ASK CAREGIVERS ONLY]** How would you rate the satisfaction of the person you care for with the aspects of the current medication for ALS, listed below?
Please use a scale from 1 to 5, where 1 is "Not at all" and 5 is "Very much".

Code	**Satisfaction**	**Rate from 1 to 5 [SLIDE BAR 1 = NOT SATISFIED AT ALL - 5 = VERY MUCH]**
1	Satisfaction with the formulation	**[SLIDE BAR 1-5]**
2	Satisfaction with handiness and easiness of preparing the treatment before administration	**[SLIDE BAR 1-5]**
3	Satisfaction with the speed in which the medication acts	**[SLIDE BAR 1-5]**
4	Satisfaction with easiness to self-administer	**[SLIDE BAR 1-5]**
5	Satisfaction with package weight and size	**[SLIDE BAR 1-5]**

B130. **[ASK PATIENTS ONLY]** Thinking about a hypothetical product containing the attributes listed below, how important are each of the aspects listed below, for you?
[ASK CAREGIVERS ONLY] Thinking about a hypothetical product containing the attributes listed below, how important are each of the aspects listed below, for the person with ALS you care for?
Please indicate your response on a scale from 1 to 5, where 1 is "Not at all" and 5 is "Very much".

Code	Compelling aspects of hypothetical products	Rate from 1 to 5 [SLIDE BAR 1 = NOT AT ALL - 5 = VERY MUCH]
1	It can dissolve in the mouth without the need to engage the tongue	[SLIDE BAR 1-5]
2	No need for water and no need to stimulate salivation	[SLIDE BAR 1-5]
3	Reduced risk of contamination* compared with available treatments	[SLIDE BAR 1-5]
4	Reduced risk of underdosing** compared with available treatments	[SLIDE BAR 1-5]
5	Reduced metallic taste compared with available treatments	[SLIDE BAR 1-5]
6	Potential self-administration and independence	[SLIDE BAR 1-5]
7	Intuitive and easy use without the need for extensive instructions	[SLIDE BAR 1-5]
8	Convenient and portable packaging	[SLIDE BAR 1-5]

[ADD DEFINITION AT BOTTOM OF B130] ***Risk of *contamination***: *Patient or caregiver risk of adding unwanted contaminant materials in the treatment due to tablet crushing, such as dirt or bacterial agents*
[ADD DEFINITION AT BOTTOM OF B130] ****Risk of *underdosing***: *Patient or caregiver risk of not administering the recommended dosage of the treatment due to tablet crushing*

B140. [ONLY IF S90R4 NOT SELECTED] [ASK PATIENTS ONLY] When thinking about changing medication, which of the reasons listed below best describe why you would want to change medication?
[ONLY IF S90R4 NOT SELECTED]
[ASK CAREGIVERS ONLY] When thinking about changing medication, which of the reasons listed below best describe why the person you care for would want to change medication?
[ONLY IF S90R4 NOT SELECTED] [SHOW ALL] *Please rank the reasons from 1 to 7, where 1 is the most important reason and 7 the least.*

Code	**Importance of reasons to switch** [RANDOMIZE; KEEP OTHER AT BOTTOM]	**Multiselect** [RATE SELECTION FROM 1 TO 7; DO NOT ALLOW TO PROCEED IF < 7 OR > 7 ARE SELECTED; NO UPPER LIMIT IF OTHER IS SELECTED]
1	If I need a gastrostomy in the future	□
2	If it facilitates ingesting the treatment	□
3	If it has less risk of choking	□
4	If I can take/continue taking it on my own	□

5	If it allows me to stay with the same treatment in the future because of how convenient it is to take	◻
6	At my caregiver's or family's request	◻
7	If my doctor recommends it	◻
99	Other, please specify:______ **[ENABLE FREE TEXT]**	◻

SECTION C: DISCRETE CHOICE EXPERIMENT WITHOUT ROF – [DO NOT SHOW SECTION HEADER]

For the remaining part of the survey, you will be shown 10 pairs of cards. Each card will represent a hypothetical medication for ALS and its features. Please click on the card that you would choose as a treatment for ALS given a choice between the two medications.
[DO NOT SHOW TABLE OF ATTRIBUTES BELOW TO RESPONDENTS: EACH ATTRIBUTE LEVEL BELOW TO BE MIXED IN ORDER TO CREATE RANDOM CARDS WITH DIVERSE PRODUCTS]

Attributes ranked by importance	Level 1	Level 2	Level 3
**Treatment administration** Administration of treatment to the patient	I can administer this treatment to myself	I can administer this treatment to myself but with the support of my caregiver	I cannot administer this treatment to myself, my caregiver needs to administer it to me
**Ease of swallowing** Ways in which a treatment may or may not interfere with a patient's swallowing process	There is no swallowing discomfort as no swallowing is needed for this treatment	There is a potential swallowing discomfort due to this treatment formulation	There is a significant swallowing discomfort due to this treatment formulation
**Mouth numbness (e.g., tingling)** Temporary local anesthetic effect (i.e. loss of sensation) of the treatment in the mouth	There is no mouth numbness (e.g. tingling) with this treatment	There is ~40% risk of experiencing mouth numbness (e.g., tingling) with this treatment, for a few minutes	There is >40% risk of experiencing mouth numbness (e.g., tingling) with this treatment, for a few minutes
**Treatment taste** Taste linked to the treatment	This treatment has no taste	There is a metallic taste associated with this treatment	---
**Treatment manipulation** Need to manipulate or change of a treatment formulation by a patient or caregiver to facilitate administration	There is no manipulation needed with this treatment	Treatment manipulation can help with the administration of this treatment e.g. crushing and/or mixing with food or liquid	---
**Formulation** Treatment formulation	This treatment is a tablet that should be swallowed: the size is comparable to a pine nut that needs to be swallowed	This treatment is an oral suspension (i.e. liquid) comparable to a liquid thicker than honey that needs to be swallowed or given via feeding tube	---

FOR MARKET RESEARCH PURPOSES ONLY

C10. Which of the following products do you prefer?

[PLEASE LOOP 10X TO SHOW 10 PAIRS OF 2 CARD CHOICES]

SECTION D: DISCRETE CHOICE EXPERIMENT WITH ROF – [DO NOT SHOW SECTION HEADER]

We will now repeat the previous exercise but with added attributes. Please click on the card that you would choose as a treatment for ALS given a choice between the two medications.
[DO NOT SHOW TABLE OF ATTRIBUTES BELOW TO RESPONDENTS: EACH ATTRIBUTE LEVEL BELOW TO BE MIXED IN ORDER TO CREATE RANDOM CARDS WITH DIVERSE PRODUCTS]

Attributes ranked by importance	Level 1	Level 2	Level 3
**Treatment administration** Administration of treatment to the patient	I can administer this treatment to myself	I can administer this treatment to myself but with the support of my caregiver	I cannot administer this treatment to myself, my caregiver needs to administer it to me
**Ease of swallowing** Ways in which a treatment may or may not interfere with a patient's swallowing process	There is no swallowing discomfort as no swallowing is needed for this treatment	There is a potential swallowing discomfort due to this treatment formulation	There is a significant swallowing discomfort due to this treatment formulation
**Mouth numbness (e.g., tingling)** Temporary local anesthetic effect (i.e. loss of sensation) of the treatment in the mouth	There is no mouth numbness (e.g. tingling) with this treatment	There is ~40% risk of experiencing mouth numbness (e.g., tingling) with this treatment, for a few minutes	There is >40% risk of experiencing mouth numbness (e.g., tingling) with this treatment, for a few minutes
**Treatment taste** Taste linked to the treatment	This treatment has no taste	There is a metallic taste associated with this treatment	---
**Treatment manipulation** Need to manipulate or change of a treatment formulation by a patient or caregiver to facilitate administration	There is no manipulation needed with this treatment	Treatment manipulation can help with the administration of this treatment e.g. crushing and/or mixing with food or liquid	---
**Formulation** Treatment formulation	This treatment is a tablet that should be swallowed: the size is comparable to a pine nut that needs to be swallowed	This treatment is an oral suspension (i.e. liquid) comparable to a liquid thicker than honey that needs to be swallowed or given via feeding tube	This treatment is an oral film that is taken via the mouth as a strip which dissolves upon contact with the tongue

D10. Which of the following products do you prefer?

[PLEASE LOOP 10X TO SHOW 10 PAIRS OF 2 CARD CHOICES]

SECTION E: PATIENT REPORTED OUTCOME & QOL – [DO NOT SHOW SECTION HEADER]

B10. [SHOW ALL] Please indicate to your best knowledge when you first started experiencing ALS symptoms.

Code	Symptom onset	
1	Month [ALLOW FOR “I DON’T KNOW”]	[PROGRAMM DROPDOWN MENU IN MM/YYYY FORMAT]
2	Year [RANGE 1922-2022]	

B20. [ASK PATIENTS ONLY] When were you diagnosed with ALS?
[ASK CAREGIVERS ONLY] When was the person you care for diagnosed with ALS?

Code	Diagnosis time [DO NOT ALLOW FOR DATE BEFORE B10R1 & B10R2]	
1	Month [ALLOW FOR “I DON’T KNOW”]	[PROGRAMM DROPDOWN MENU IN MM/YYYY FORMAT]
2	Year [RANGE 1900-2022]	

B103. [ONLY IF B101R2>0] [ASK PATIENTS ONLY] You mentioned that you can experience difficulties in taking your treatment or you may need a caregiver to assist you in taking your medication. Which of these most accurately reflects how you feel about taking your medicine?
[ONLY IF B101R2>0] [ASK CAREGIVER ONLY] You mentioned that the person with ALS you care for can experience difficulties in taking their treatment or they may need a caregiver to assist in taking their medication. Which of these most accurately reflects how they feel about taking your medicine?

Code	Patient feeling about treatment	Multiselect
1	It can sometimes be embarrassing or humiliating	□
2	I feel it takes away some of my dignity	□
3	I don’t feel embarrassed at all	□ [IF B103R1 SELECTED, SHOW ERROR MESSAGE AS B103R1 &

		B103R3 CANNOT BE SELECTED TOGETHER]

B150. **[ASK PATIENTS ONLY]** Please indicate below how the treatment decision making with your doctor looks like, when it comes to the formulation.
[ASK CAREGIVERS ONLY] Please indicate below how the treatment decision making with the doctor of the person with ALS you care for looks like, when it comes to the formulation.

Code	1	[INSERT SLIDER FROM 1 TO 7]	7
1	I made the choice of formulation alone with little to no input from my doctor		My doctor made the choice of formulation alone with little to no input from me

E10. **[ASK PATIENTS ONLY]** Thinking about the past week (7 days), how was your wellbeing?
[ASK CAREGIVERS ONLY] Thinking about the past week (7 days), how was wellbeing of the person you care for?
Please indicate your response on a scale from 1 to 5, where 1 is "Very bad" and 5 is "Excellent".

Code	**Wellbeing**	**Rate from 1 to 5 [SLIDE BAR 1 = VERY BAD - 5 =EXCELLENT]**
1	Considering all parts of my life – physical, emotional, social, spiritual, and financial – over the past week, my wellbeing has been	**[SLIDE BAR 1-5]**

E20. **[ASK PATIENTS ONLY]** Thinking about your personal day to day experience with ALS, how much do you relate/agree to the following statements?
[ASK CAREGIVERS ONLY] Thinking about your personal day to day experience with ALS, how much do you relate/agree to the following statements?
*Please indicate your response as it applies to the **past month**, on a scale from 1 to 5, where 1 is "Not at all" and 5 is "Very much".*

Code	**Wellbeing**	**Rate from 1 to 5 [SLIDE BAR 1 = NOT AT ALL - 5 = VERY MUCH]**
1	I am hopeful in the fight against my illness	**[SLIDE BAR 1-5]**

2	I feel in control of my condition	[SLIDE BAR 1-5]
3	I have accepted my condition	[SLIDE BAR 1-5]
4	I feel productive	[SLIDE BAR 1-5]
5	I have felt embarrassed in social situations	[SLIDE BAR 1-5]
6	I have worried that I am a burden to other people	[SLIDE BAR 1-5]

E30. **[ASK PATIENTS ONLY]** Please rate the following symptoms and experiences according to how much of a problem each one has been for you.
[ASK CAREGIVERS ONLY] Please rate the following symptoms and experiences according to how much of a problem each one has been for the person you care for.
Please respond about how you have felt or what you have experienced over the past week on a scale from 1 to 5, where 1 is "No Problem" and 5 is "Tremendous problem"

Code	**Symptoms and experiences**	**Rate from 1 to 5 [SLIDE BAR 1 = NO PROBLEM - 5 = TREMENDOUS PROBLEM]**
1	Pain	[SLIDE BAR 1-5]
2	Fatigue	[SLIDE BAR 1-5]
3	Eating	[SLIDE BAR 1-5]
4	Excess Saliva	[SLIDE BAR 1-5]
5	Mucous in Throat	[SLIDE BAR 1-5]
6	Speaking	[SLIDE BAR 1-5]
7	My strength and ability to move	[SLIDE BAR 1-5]
8	Sleep	[SLIDE BAR 1-5]
9	Bowel and Bladder (Constipation, Diarrhea, Poor Control)	[SLIDE BAR 1-5]

E40. **[ASK PATIENTS ONLY]** We will now ask you to select between statement relating to your salivation
[ASK CAREGIVERS ONLY] We will now ask you to select between various statement relating to the salivation of the ALS of the person you care for
[SHOW ALL] Please select below all statements that best apply to your ALS.

Code	**Category [RANDOMIZE CATEGORY]**	**Functional ALS rating**	**Single select per category**

Salivation [KEEP CATEGORY ELEMENTS IN ORDER]			
1		Normal	☐
2		Light but definite excess of saliva in mouth, may have night-time drooling	☐
3		Moderately excessive saliva; may have minimal drooling (during the day)	☐
4		Marked excess of saliva with some drooling	☐
5		Marked drooling; requires constant tissue or handkerchief	☐

E41. Below, you will find some general statements that people with swallowing problems might mention. Please indicate, in the **last month,** how true these statements have been for you/the person with ALS you care for.
Please rate your answers from 1 to 5, where 1 is "Not at all true" and 5 is "Totally true"

Code	**Swallowing statements**	**Rate from 1 to 5 [SLIDE BAR 1 = NOT AT ALL TRUE - 5 = TOTALLY TRUE]**
1	Dealing with my swallowing problem is very difficult	**[SLIDE BAR 1-5]**
2	My swallowing problem is a major distraction in my life	**[SLIDE BAR 1-5]**
3	I fear I may start choking when I eat food	**[SLIDE BAR 1-5]**
4	I worry about getting pneumonia	**[SLIDE BAR 1-5]**
5	I am afraid of choking when I drink liquids	**[SLIDE BAR 1-5]**
6	I never know when I am going to choke	**[SLIDE BAR 1-5]**
7	My swallowing problem depresses me	**[SLIDE BAR 1-5]**
8	Having to be so careful when I eat or drink annoys me	**[SLIDE BAR 1-5]**
9	I've been discouraged by my swallowing problem	**[SLIDE BAR 1-5]**
10	My swallowing problem frustrates me	**[SLIDE BAR 1-5]**
11	I get impatient dealing with my swallowing problem	**[SLIDE BAR 1-5]**

[SHOW IN A NEW PAGE]
Thank you for your participation in this survey. In connection with CRA, Zambon S.p.A. is a joint controller of your personal information. Their full privacy notice is available here https://www.zambon.com/en/privacy-policy#:~:text=Personal%20data%20is%20processed%20on,which%20the%20data%20wa

s%20collected.&text=Zambon%20S.p.A.%20holds%20no%20responsibility,data%20outside%20of%20its%20control

Now that the Sponsor of the Project has been revealed, if you wish to withdraw your consent and/or no longer wish to participate, please tick this box. If you withdraw consent, we will delete any personal data that was collected from you.

	**Sponsor**	**Select one**
1	I give consent and wish to participate	☐ **[CONTINUE]**
2	I withdraw consent. My data will be deleted	☐ **[TERMINATE]**

This concludes the survey. Thank you very much for taking the time. Do you have any final comments or remarks about any of the topics we asked you about or any feedback on the survey?

**[ENABLE FREE TEXT]**

Thank you and have a good day.
[END OF SURVEY]
