# Supplementary material for: The preferences of people with amyotrophic lateral sclerosis on riluzole treatment in Europe
Source: Sci Rep. 2023 Dec 15;13:22497. doi: 10.1038/s41598-023-49424-3 (PMC10728064; doi:10.1038/s41598-023-49424-3)
Supplement: Supplementary file 2 — Supplementary Information 2. [file 41598_2023_49424_MOESM2_ESM.docx]

| **Participant demographics and clinical characteristics** | **Survey, Overall (N=109)** | **Qualitative interview (N=8)** |
| --- | --- | --- |
| **Nation (n)** |  |  |
| Germany | 30 | 2 |
| Italy | 30 | 2 |
| Spain | 47 | 2 |
| France | 2 | 2 |
| **Patients (n)** | 54 | 4 |
| **Caregivers (n)** | 55 | 4 |
| **Age (n)** |  |  |
| 20-29 years | 2 |  |
| 30-39 years | 12 |  |
| 40-49 years | 27 |  |
| 50-59 years | 36 |  |
| 60-69years | 25 |  |
| 70+ years | 7 |  |
| **Time from symptom onset to diagnosis (mean in months)** | 12.5 |  |
| **Time since diagnosis until time of analysis (mean in years)** | 4.7 |  |
| **ALS type of onset (n, %)** |  |  |
| Spinal onset | 68 , 62 |  |
| Bulbar onset | 19, 17 |  |
| Mixed onset | 19, 17 |  |
| Not known | 3, 3 |  |
| **Patients w/ dysphagia (n,%)** | 76, 70 |  |
| **Patients w/o dysphagia (n,%)** | 33, 30 |  |
| **Feeding needs (n, %)** |  |  |
| Normal eating habits | 33, 30 |  |
| Problems with feeding, possible choking | 20, 18 |  |
| Changes in food consistency (provoked by difficulty in swallowing) | 38, 35 |  |
| Supplement tube for feeding required | 7, 6 |  |
| Complete dependence on feeding tube | 11, 10 |  |
| **Functionality (n, %)** |  |  |
| **Speech:** |  |  |
| able to communicate intelligibly | 33, 30 |  |
| loss of useful speech | 76, 70 |  |
| **Typewriting:** |  |  |
| able to type on a keyboard /computer | 40, 37 |  |
| not able to write | 69, 63 |  |
| **Eating habits:** |  |  |
| not have a gastrostomy and able to feed without help | 24, 22 |  |
| not have a gastrostomy but need help with feeding | 54, 50 |  |
| have a gastrostomy and need minimal assistance | 8, 7 |  |
| have a gastrostomy and I need significant assistance | 23, 21 |  |
| **Dressing and hygiene:** |  |  |
| independent in dressing and hygiene | 18, 17 |  |
| need significant assistance in self-care | 91, 83 |  |

**Supplementary Table 1** Participant demographics and clinical characteristics

| **Patient treatment history and behaviors** | **Survey, Overall (N=109)** |
| --- | --- |
| **Clinical trial participation (n, %):** |  |
| Yes | 11, 10 |
| Pharmaceutical treatment | 9, 82 |
| Non-Pharmaceutical treatment | 1, 9 |
| I don’t know | 1, 9 |
| No | 98, 90 |
| **Previous medication type (n, %):** |  |
| riluzole (tablet formulation) | 73, 67 |
| riluzole (oral suspension formulation) | 32, 29 |
| edaravone (intravenous) | 5, 5 |
| no treatment | 0 |
| other [mexiletine, dextromethorphan-quinidine, gabapentin, lorazepam, baclofen, metamizole] | 6, 6 |
| **Current medication (n,%):** |  |
| ALS-specific treatment | 108, 99 |
| Any ALS symptomatic treatment | 1, 1 |
| **Switching or interrupting treatment (%)** | 13 |
| **Reason for switching (n)*:** | 8 responders |
| did not like the taste of the medicine | 0 |
| did not like the feeling in the mouth / mouth numbness | 0 |
| had difficulty swallowing the tablet formulation | 4 |
| had difficulty swallow the oral suspension / thick liquid | 0 |
| did not like carrying bottles and syringes when travelling | 0 |
| did not like crushing the tablets | 0 |
| My doctor decided to change the treatment | 1 |
| Other reasons [reported as: side effects experienced, like vertigo or perception that the condition keeps progressing] | 5 |

**Supplementary Table 2** Patient treatment history and changes in treatment.*8 PALS reported the reason for switching (multiple choice allowed).


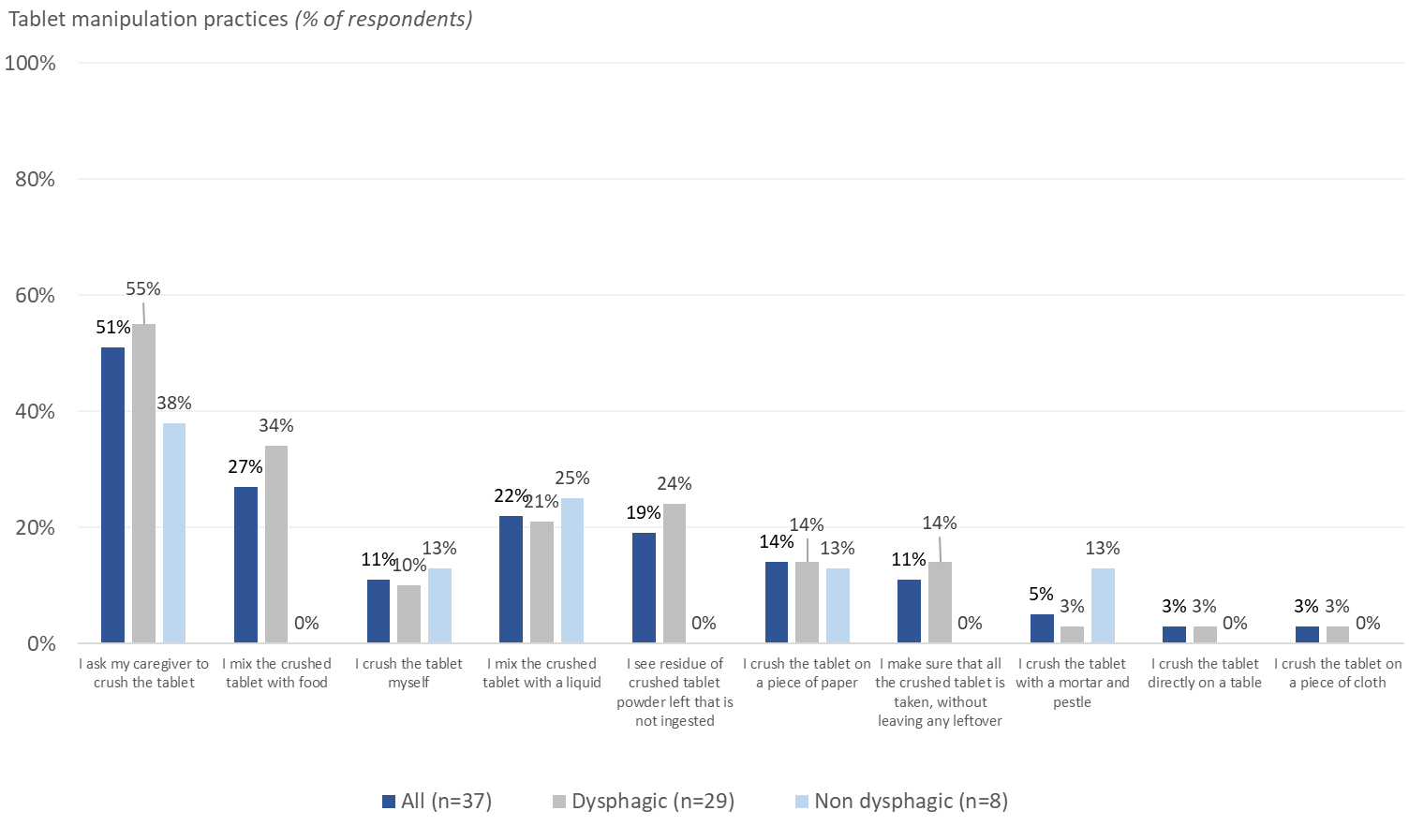


**Supplementary Figure 1** Tablet manipulation methods practiced by ALS patients. All percentages were calculated versus the respondents per each group. The question allowed multiple answers.


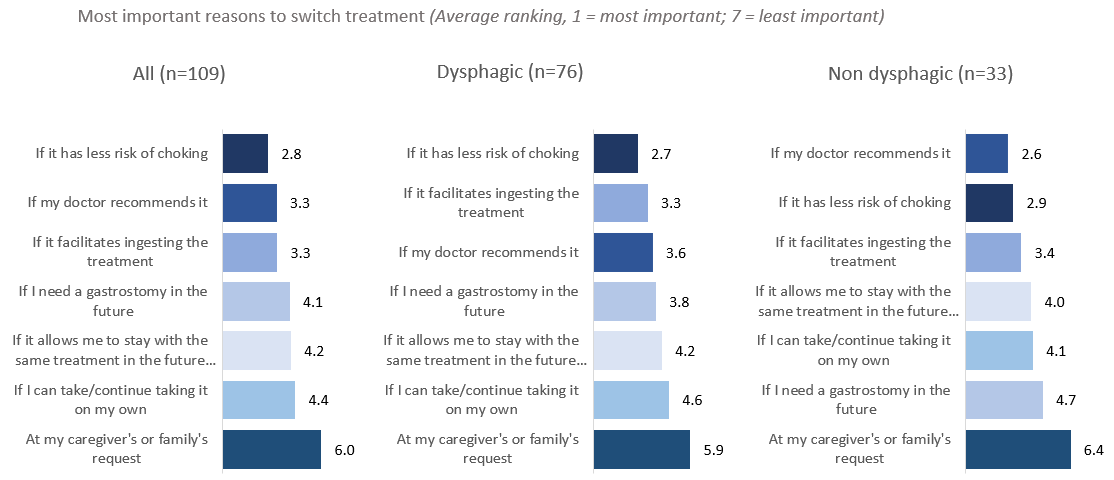


**Supplementary Figure 2** Reasons for switching the treatment. All percentages were calculated versus the respondents per each group


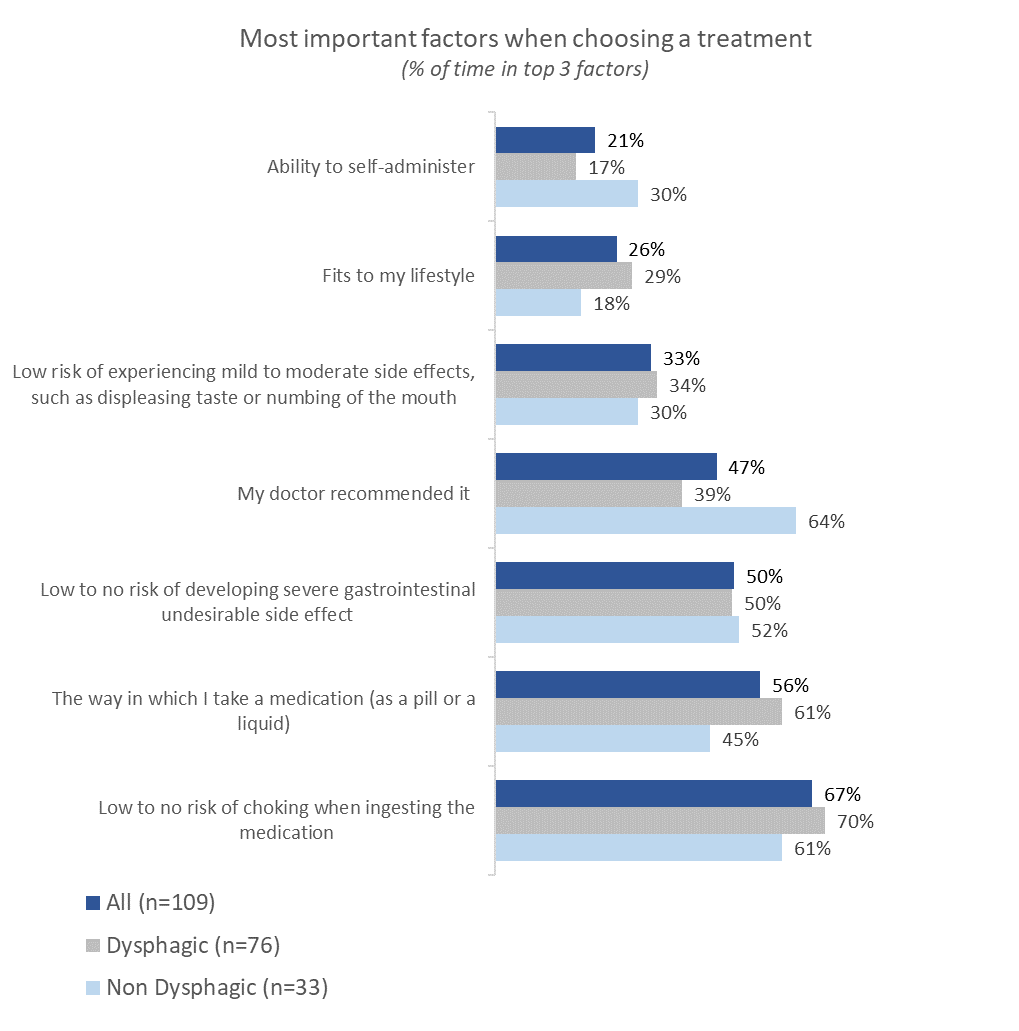


**Supplementary Figure 3** Most important factors when choosing a treatment. All percentages were calculated versus the respondents per each group

| **Level of compellingness of characteristics of new oral film formulation** | **Tablet users** | | | | **Oral suspension users** | | | |
| --- | --- | --- | --- | --- | --- | --- | --- | --- |
| **Frequency of manipulation** | **Always crush** | **Sometimes crush** | **No crush** | **Mean** | **Always mix** | **Sometimes mix** | **No mix** | **Mean** |
| Intuitive and easy use without the need for extensive instructions | 4.2 | 4.3 | 4.4 | 4.3 | 5.0 | 3.4 | 4.0 | 4.1 |
| Convenient and portable packaging | 4.1 | 4.0 | 4.0 | 4.0 | 3.2 | 3.9 | 4.2 | 3.8 |
| Potential self-administration and independence | 3.5 | 3.7 | 4.3 | 3.9 | 5.0 | 3.1 | 3.6 | 3.9 |
| It can dissolve in the mouth without the need to engage the tongue | 3.9 | 3.0 | 4.1 | 3.8 | 5.0 | 3.3 | 4.1 | 4.0 |
| No need for water and no salivary stimulant | 3.8 | 2.7 | 4.1 | 3.8 | 4.9 | 3.6 | 3.5 | 4.0 |
| Reduced risk of underdosing compared with available treatments | 4.1 | 3.1 | 3.6 | 3.7 | 4.4 | 4.1 | 3.7 | 4.0 |
| Reduced risk of contamination compared with available treatments | 4.0 | 3.0 | 3.6 | 3.7 | 4.2 | 3.7 | 3.5 | 3.8 |
| Reduced metallic taste compared with available treatments | 3.7 | 3.6 | 3.4 | 3.6 | 4.8 | 2.9 | 4.1 | 4.0 |

**Supplementary Table 3** Level of appealing new formulation attributes according to the frequency of alteration in tablet and oral suspension users. Patients reported the compellingness on a scale from 1 (not compelling, left side) to 5 (very compelling, right side)
